# An ovine hepatorenal fibrocystic model of a Meckel-like syndrome associated with dysmorphic primary cilia and *TMEM67* mutations

**DOI:** 10.1038/s41598-017-01519-4

**Published:** 2017-05-09

**Authors:** C. Stayner, C. A. Poole, S. R. McGlashan, M. Pilanthananond, R. Brauning, D. Markie, B. Lett, L. Slobbe, A. Chae, A. C. Johnstone, C. G. Jensen, J. C. McEwan, K. Dittmer, K. Parker, A. Wiles, W. Blackburne, A. Leichter, M. Leask, A. Pinnapureddy, M. Jennings, J. A. Horsfield, R. J. Walker, M. R. Eccles

**Affiliations:** 1 Department of Pathology, Dunedin School of Medicine, University of Otago, PO Box 56 Dunedin 9054, New Zealand; 2 Department of Medicine, Dunedin School of Medicine, University of Otago, PO Box 56 Dunedin 9054, New Zealand; 3 Department of Anatomy and Medical Imaging, The University of Auckland 1142, Private Bag, 92019 Auckland, New Zealand; 4 AgResearch Invermay Agricultural Centre, Mosgiel, 9053 New Zealand; 5 Institute of Veterinary, Animal and Biomedical Sciences, Massey University, Tennant Drive, Palmerston North, 4472 New Zealand; 6 150 Warren Street, Wanaka, 9305 New Zealand

## Abstract

Meckel syndrome (MKS) is an inherited autosomal recessive hepatorenal fibrocystic syndrome, caused by mutations in *TMEM67*, characterized by occipital encephalocoele, renal cysts, hepatic fibrosis, and polydactyly. Here we describe an ovine model of MKS, with kidney and liver abnormalities, without polydactyly or occipital encephalocoele. Homozygous missense p.(Ile681Asn; Ile687Ser) mutations identified in ovine *TMEM67* were pathogenic in zebrafish phenotype rescue assays. Meckelin protein was expressed in affected and unaffected kidney epithelial cells by immunoblotting, and in primary cilia of lamb kidney cyst epithelial cells by immunofluorescence. In contrast to primary cilia of relatively consistent length and morphology in unaffected kidney cells, those of affected cyst-lining cells displayed a range of short and extremely long cilia, as well as abnormal morphologies, such as bulbous regions along the axoneme. Putative cilia fragments were also consistently located within the cyst luminal contents. The abnormal ciliary phenotype was further confirmed in cultured interstitial fibroblasts from affected kidneys. These primary cilia dysmorphologies and length control defects were significantly greater in affected cells compared to unaffected controls. In conclusion, we describe abnormalities involving primary cilia length and morphology in the first reported example of a large animal model of MKS, in which we have identified *TMEM67* mutations.

## Introduction

Inherited renal cystic/fibrocystic diseases constitute an important subset of monogenic disorders, transmitted as autosomal dominant, autosomal recessive, or X-linked traits, and are responsible for more than 5% of worldwide end-stage renal disease^1^. Whereas the development of fluid-filled cysts and progressive impairment of renal function are common features, these disorders are distinguished from each other by different ages of onset, variable rates of renal disease progression, and a diverse array of extra-renal manifestations^1–3^. The two major types of polycystic kidney disease (PKD) in humans have autosomal dominant (ADPKD) and autosomal recessive (ARPKD) inheritance^1, 4^. ADPKD is the most common dominant genetic disease in humans, affecting 1 in 500 individuals^1^ and has a late onset. Recessive disorders include ARPKD^5^, nephronophthisis^6^, Meckel syndrome^7–9^, Joubert syndrome, Bardet-Biedl syndrome and other related disorders^10^. While less common than ADPKD, these other recessive disorders develop at a much earlier age, and generally involve a more severe disease phenotype with reduced survival^1, 5^. Several recent discoveries indicate that the aetiology of PKD is associated with structural and/or functional defects in epithelial primary cilia^4, 6, 8^, collectively termed ciliopathies^4^.

The primary cilium is a single cytoplasmic organelle found in virtually all vertebrate cells^11, 12^. It consists of two parts, a membrane-coated axoneme with a 9 + 0 microtubular doublet symmetry that projects from the cell surface into the extracellular microenvironment, and an intracellular basal body that comprises the more mature of the two centrioles located within the centrosome. The centrosome represents the microtubule organising centre of the cell, and assembly of the microtubular network is essential for the differentiation of the Golgi apparatus into functional *cis*, *medial* and *trans* compartments^13^. In renal epithelial cells the cilium projects into the lumen of the nephron and is mechanically deflected by urine flow, transducing biomechanical and physicochemical information into cellular regulatory signals^14^. Consequently, failure of the intraflagellar transport mechanism, required to assemble a primary cilium and to insert functional ciliary proteins into the axoneme, results in abnormal signal transduction, epithelial cell proliferation and renal cystogenesis^15^.

Many PKD-associated proteins have been localised to the primary cilium, or the basal body^16^. The earliest PKD mouse model to be linked with a primary cilia defect was the *TgN737Rpw* mouse, which carries a disruption in the *TgT737* gene encoding the intraflagellar transport protein IFT88/Polaris^17^. Unlike the *TgN737Rpw* phenotype, in which cilia are severely stunted, most defects in PKD-associated proteins result in the disruption of protein trafficking or cell signaling in the cilia, rather than a complete absence of structure^16^.

Meckel syndrome (MKS; OMIM #249000 and #607361), an embryonic lethal disorder with phenotypic and genetic heterogeneity, overlaps with other viable ciliopathies such as Joubert syndrome, nephronophthisis and Bardet-Biedl syndrome^18^. MKS is characterised by occipital encephalocoele, bilateral renal polycystic fibrodysplasia, hepatic fibrosis, hepatic developmental defects, biliary dysgenesis, and bilateral postaxial polydactyly^19^. Renal manifestations include massively enlarged kidneys with extensive cystogenesis of the cortex and medulla, and extensive interstitial fibrosis^20^. Renal cysts in MKS contain columnar, cuboidal and squamous epithelia with primary cilia of variable lengths^21^. The incidence of MKS is variable, with the highest incidence (1:1300) in Gujarati Indians^22^, but also high frequencies reported in North Africa (1:3500) and Finland (1:9000). In the USA, the incidence is 1:13250^22^. Mutations in any one of ten different genes have so far been linked to MKS including (other designated names); *MKS1* (*BBS13*); *MKS2* (*TMEM216*, *JBTS2*); *MKS3* (*TMEM67*, *JBTS6*, *NPHP11*); *MKS4* (*CEP290*, *BBS14*, *NPHP6*, *JBTS5*, *SLSN6*, *LCA10*); *MKS5* (*RPGRIP1L*, *NPHP8*, *JBTS7*, *FTM*); *MKS6* (*CC2D2A*, *JBTS9*); *MKS7* (*NPHP3*); *MKS8* (*TCTN2*, *TECT2*); *MKS9* (*B9D1*, *MKSR1*), *MKS10* (*B9D2*, *MKSR2*, *STUMPY*)^23, 24^. Most, if not all, MKS proteins are localised to the centrosome/basal body, the pericentriolar region, the alar sheets (transitional fibres), the transition zone, or the cilium itself^25^. Widespread distribution of the affected proteins involving primary cilia throughout most organ systems may explain the wide variety of phenotypes that are present in addition to renal cystic disease.

The *TMEM67* gene was first identified from positional cloning of the rat *Wpk* (Wistar polycystic kidney) disease locus, and linkage to human *MKS3* ^9^. *Wpk* rats are viable and exhibit polycystic kidney disease, abnormalities of the corpus callosum ranging from hypoplasia to agenesis, and severe hydrocephalus, but lack biliary abnormalities^9, 26^. Spontaneous disruption affecting the murine *Tmem67* locus was identified in *bpck* (bilateral polycystic kidney disease) mice^7^. Although viable, these mice display severe rapidly progressing renal cystic dysplasia, and hydrocephalus leading to death by 3 weeks of age. More recent studies in this animal model have revealed abnormal trafficking of some proteins into the cilium due to absence of a functional *Tmem67* gene^27^. Additionally, a second *Tmem67* knockout mouse model, *Tmem67* ^tm1(Dgen/H)^, has been generated that more closely resembles human MKS3, with renal cysts, hepatic ductal plate defects^28^, and a range of neurodevelopmental defects^29^.

Although much has been learned from studies of mice and zebrafish animal models of MKS3^7, 27–30^, large animal models of disease are thought to be generally more physiologically similar to humans, and therefore should yield experimental data more comparable to humans, particularly in terms of experimental therapeutic strategies. In addition, spontaneous pathogenic mutations occurring in large animal models may provide useful information on genotype/phenotype relationships.

Here we describe the characterisation of an ovine model, representing to our knowledge the first description of a large animal model of a Meckel-like hepatorenal fibrocystic dysplasia syndrome. In this model we have found mutations in *TMEM67*, dysmorphic primary cilia, and the presence of primary cilia fragments in cyst lumens.

## Results

### Hepatorenal fibrocystic disease and multiple additional abnormalities were observed in affected lambs

**Figure 1:**
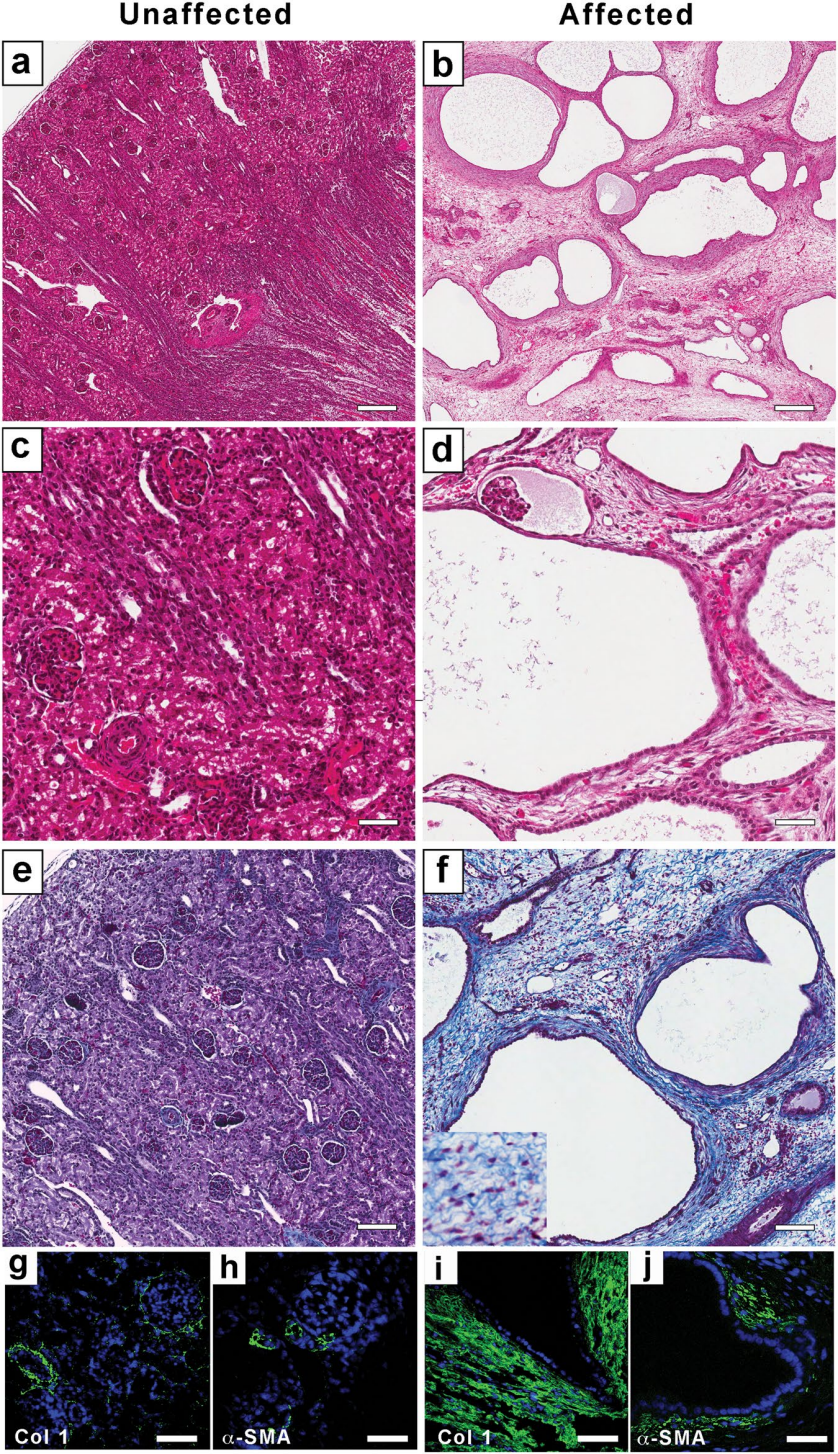
Renal fibrocystic dysplasia in affected lambs. H&E stained tissue sections at lower (**a**,**b**) and higher (**c**,**d**) magnification showing kidney architecture of an unaffected newborn lamb (**a** and **c**), compared to kidney of an affected newborn lamb, with multiple, fluid-filled cysts (1–5 mm diameter) evident throughout the cortex and medulla (**b** and **d**). Masson’s Trichrome stained sections of unaffected (**e**), and affected (**f**) kidneys. The extensive fibrosis present in the affected kidney (**f**-inset) shows thick collagen fibres (stained blue) within the interstitium. (**g–i**) Immunohistochemistry using collagen type I (green; **g**,**i**), and α-smooth muscle actin antibodies (green; **h**,**j**) on unaffected (**g**,**h**) and affected (**i**,**j**) kidneys, respectively. Nuclei (blue) are stained with DAPI. Scale bars: a, b = 200 μm; c, d, e, f = 100 μm. g, h, i, j = 50 μm.

**Figure 2:**
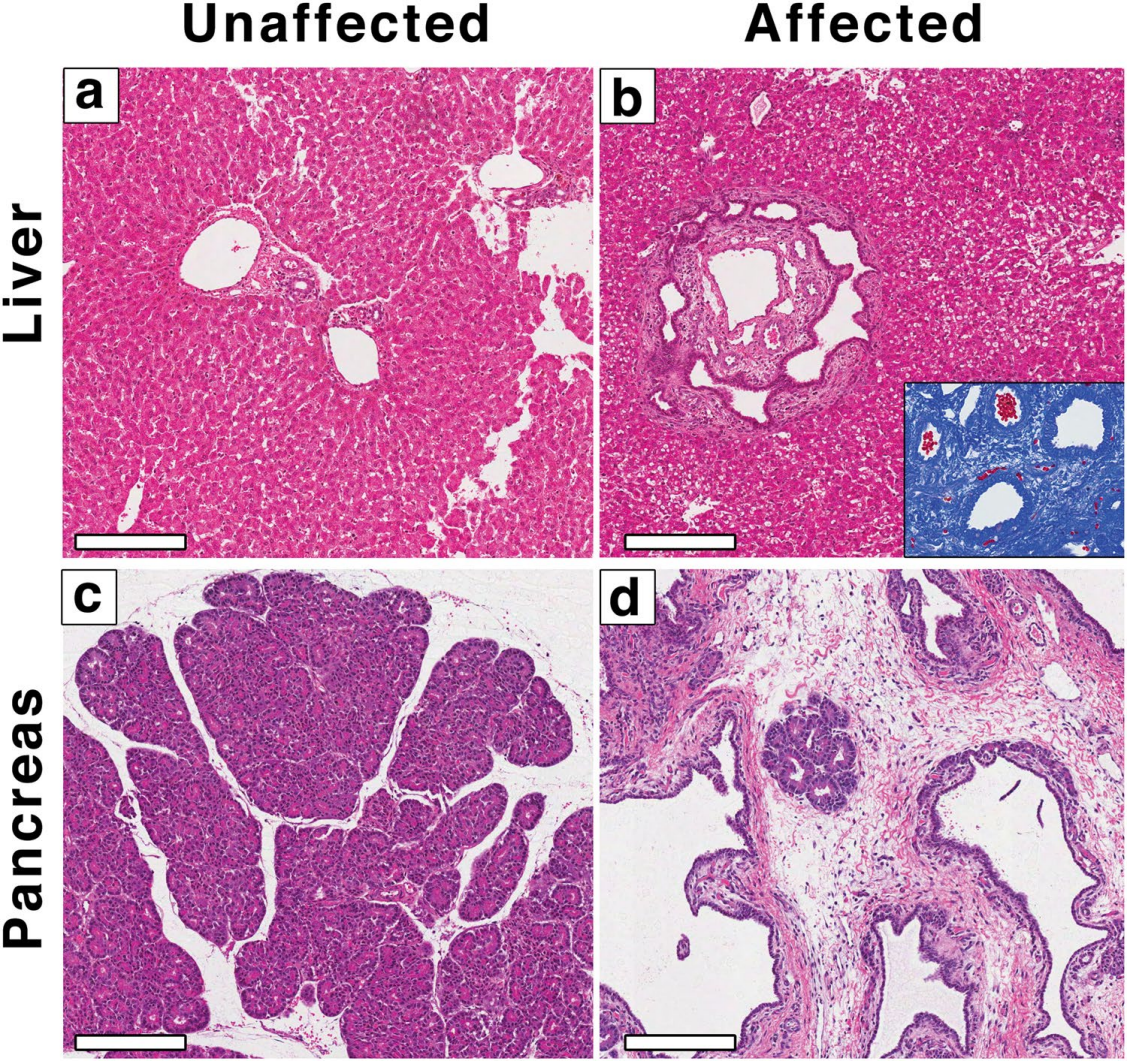
Hepatic and pancreatic abnormalities in affected lambs. (**a**,**b**) H&E stained tissue sections from an unaffected newborn lamb liver (**a**), showing the portal triad, and an affected newborn lamb liver (**b**), showing the ductal plate malformation in the liver of the affected lamb. (**b**-inset) Masson’s Trichrome stained section showing extensive fibrosis. (**c**,**d**) H&E stained tissue sections from an unaffected (**c**), and an affected (**d**) newborn lamb pancreas, showing extensive fibrosis surrounding an island of pancreatic tissue in the affected lamb. Scale bars = 200 μm.

Investigations that focused on hepatorenal fibrocystic disease in two NZ sheep flocks (designated A and B) and their offspring, affected with a congenital PKD, have been described previously^31^. In flocks A and B, affected lambs died either perinatally or within a few hours of birth, invariably with massively enlarged polycystic kidneys and liver fibrosis. Polycystic abnormalities were also frequently observed in the pancreas, epididymis and occasionally in the liver^31^. Affected kidneys showed progressive renal cyst formation (1–5 mm diameter); the renal cortical and medullary structures were not easily discernible (see Figs 2 and 8 in ref. 31, and Supplementary Figure S1), and significant interstitial fibrosis radiated from the hilum to the outer cortical regions of the kidneys^31^. In contrast to the kidneys of an unaffected lamb, fluid-filled cysts were observed in H&E-stained histological sections of affected kidneys at low magnification (Fig. 1a,b). At higher magnification, relative to the unaffected kidney cortex, epithelial cells in Bowman’s capsule and renal tubules were cystic, with evidence of flocculent material present in the cyst luminal contents (Fig. 1c,d). Masson’s trichrome staining showed more fibrosis in affected than unaffected kidneys (Fig. 1e,f), represented by dense irregular connective tissue (Fig. 1f inset). Immunohistochemistry confirmed abundant interstitial type I collagen expression and sub-populations of α-smooth muscle actin-positive cells in affected compared with unaffected kidneys (Fig. 1g,j). A ductal plate malformation in the liver was associated with incomplete ductal plate remodeling, resulting in numerous embryonic duct structures and an increase in portal fibrosis (Fig. 2a,b). Compared to unaffected lambs, extensive liver fibrosis (Fig. 2b inset) and biliary dysgenesis were also observed. Extensive fibrosis was also observed in affected pancreatic tissue, with loss of lobular organization. There were few remaining acini and ducts compared to unaffected pancreas (Fig. 2c,d).

### Shared regions of homozygosity were identified on chromosomes 9 and 11 in the affected offspring of flocks A and B by homozygosity mapping

**Figure 3:**
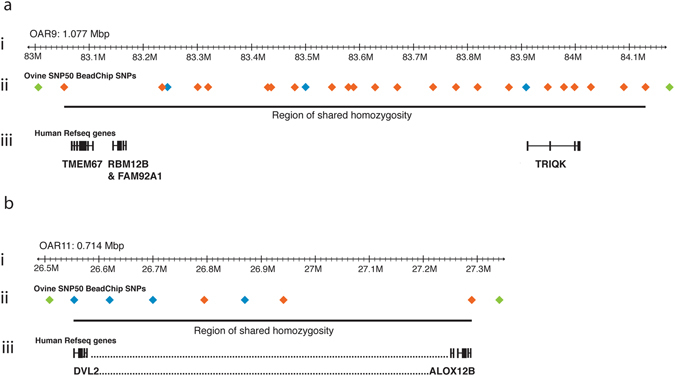
Homozygosity mapping depicting the largest homozygous regions shared by affected lambs in flocks A and B. The relevant portions of sheep chromosomes 9 and 11 are shown in (**a**) and (**b**), respectively. (ai and bi) Ruler segments depict the portion of either chromosome 9 (ai), or chromosome 11 (bi) of the ovine genome assembly v3.1 sequence. (aii and bii) colored diamonds show SNP locations on the SNP50 BeadChip in relevant regions on chromosomes 9 and 11. Green diamonds show heterozygous SNPs outside the homozygous regions. Orange diamonds show homozygous SNPs in all affected lambs. Blue diamonds show SNPs that were heterozygous in one or more affected lambs. (Note that 25 SNPs in the homozygous region in aii, and 7 SNPs in bii are depicted in this figure, but originally 26 SNPs and 8 SNPs, respectively, were identified in the v1.0 sequence - see Supplementary Tables 1–3). Regions of shared homozygosity are depicted below the SNPs by a black line. (aiii) The black boxes show the human reference sequence genes located in the regions of shared homozygosity based on comparative genomic maps of chromosomes 9 and 11. The *TMEM67* gene is indicated on chromosome 9. On chromosome 11 only two genes are shown, *DVL2* and *ALOX12B*, with the dotted line in between representing 49 other genes in this interval.

Between 2005 and 2015 carrier rams were mated with carrier ewes, and 37/307 lambs born in flocks A and B were affected with the PKD phenotype. Monogenic inheritance of an autosomal recessive trait involving PKD with reduced penetrance was inferred from the matings. Ovine SNP50 BeadChips were used to map genomic regions of shared homozygosity to delineate a genetic abnormality segregating in the two flocks. Homozygosity of genomic regions was determined in 6 affected lambs of flock A, and 14 affected lambs of flock B. Two 0.75–1 Mb homozygous genomic segments were identified on ovine chromosomes 9 and 11 in flock A (Fig. 3), which exceeded an 8-SNP threshold window for minimum concordant homozygosity, while three homozygous segments on chromosomes 4, 9 and 11 exceeded an 8-SNP threshold in flock B. In all affected lambs of both flocks, SNPs in the shared homozygous regions on ovine chromosomes 9 and 11 exhibited identity by descent (IBD).

The regions of homozygosity on chromosomes 9 and 11 were in essentially identical chromosomal locations in flocks A and B, while the homozygous region on chromosome 4 in flock B was not observed in flock A, and was therefore not investigated further. For more detailed descriptions of the homozygous regions on chromosomes 9 and 11 refer to Supplementary Tables S1 to S3, and to the additional information in the Supplementary Information.

### Affected lambs carry homozygous amino acid substitutions at the *TMEM67* locus

**Figure 4:**
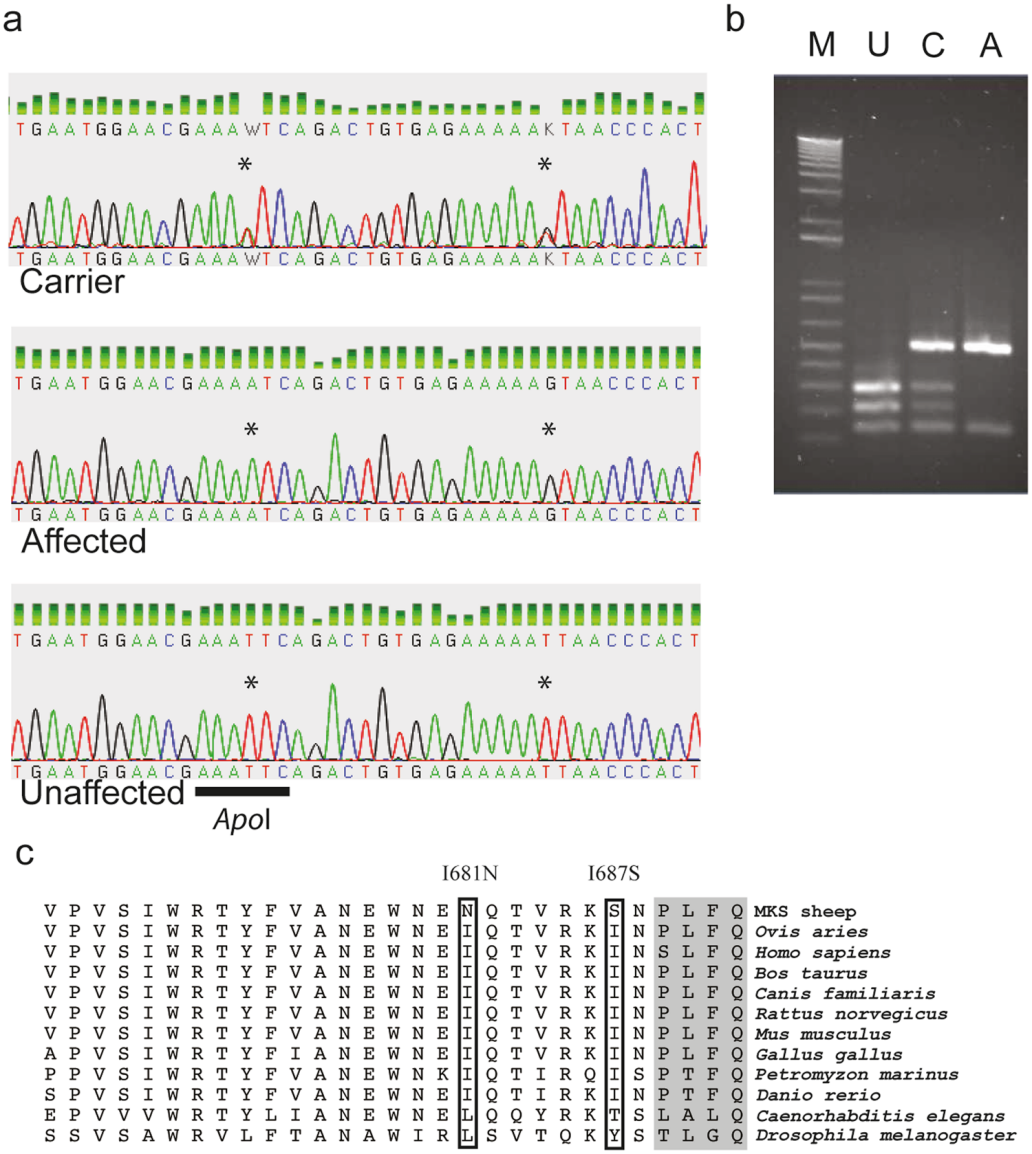
Identification of two missense mutations in *TMEM67*. (**a**) Sequence analysis of *TMEM67* showing sequence variants in exon 20 (indicated by asterisks above the sequence tracks) in a carrier, and in an affected lamb, compared to an unaffected lamb. Below the unaffected sequence, the *Apo*I restriction site is indicated (black bar) (**b**) *Apo*I restriction fragment length polymorphism generated by the sequence variants caused different restriction digestion patterns for the affected and carrier lambs, compared to unaffected lambs; M, marker; U, unaffected pattern; C, carrier pattern; A, affected pattern. (**c**) Alignment of mutant with wild type partial sequences of meckelin from multiple species. The diagram shows the isoleucine to asparagine amino acid substitution at position 681 (I681N), and isoleucine to serine substitution at position 687 (I687S) in an affected lamb (MKS sheep), compared to the unaffected (*Ovis aries*) meckelin sequence (boxed). The partial meckelin sequence is aligned with ten orthologous sequences from other species. The boxes show the affected amino acids, and conservation of an isoleucine amino acid at these positions in several vertebrate species, including humans and zebrafish. The amino acids in the grey portion mark the beginning of a transmembrane domain in meckelin.

Whole genome re-sequencing of genomic DNA from affected lamb tissues was undertaken to identify potentially pathogenic sequence variants in genes located in the homozygous regions on chromosomes 9 or 11 (refer to Supplementary Tables S4 to S6, and for additional information on the sequence variants refer to Supplementary Information). We identified two predicted non-tolerated homozygous missense sequence variants in exon 20 of the *TMEM67* gene. Three pathogenic sequence prediction programmes (A-GVGD, SIFT, and Polyphen) all suggested that both sequence variants would result in deleterious amino acid changes in the meckelin protein encoded by *TMEM67* (Supplementary Table 6). In humans, mutations in *TMEM67* have previously been associated with Meckel syndrome type 3 (MKS3). Clinical features in MKS3 closely resemble those in Meckel syndrome type 1 (caused by mutations in *MKS1*); although in MKS3, polydactyly and encephalocoele are less frequent. PCR sequencing of ovine *TMEM67* exons confirmed the presence of two sequence variants, c.2050 T > A in exon 20, and c.2068 T > G in exon 20 of *TMEM67* (refer to ovine *TMEM67* transcript in the ENSEMBL database ID: ENSOART00000009213) (Fig. 4a). The sequence variants were homozygous in all affected lambs, heterozygous in all carriers, and absent in all unaffected non-carrier sheep analysed from flocks A and B, and were also absent in > 30 sheep of varying breeds with no history of kidney disease. An *Apo*I restriction fragment length polymorphism assay was designed to detect the *TMEM67* c.2050 T > A exon 20 missense variant (Fig. 4b), and genotype assay results were concordant with phenotypic status in 40 sheep in flock B (Supplementary Table 7).

The two identified sequence variants in ovine *TMEM67* exon 20 were predicted to cause two amino acid substitutions, an isoleucine to asparagine substitution at amino acid position 681 (I681N) (Supplementary Figure S2) in the putative ovine meckelin protein (which corresponds to translated amino acid position 680 of human *TMEM67* transcript variant 1, mRNA; Refseq NM_153704.5), and an isoleucine to serine substitution at amino acid position 687 (I687S) in ovine meckelin (corresponding to amino acid position 686, in human *TMEM67*). These amino acid substitutions occurred at amino acids conserved in mammals (sheep, human, cattle, dog, rat, and mouse), as well as chicken, lamprey and zebrafish, but not conserved in flies or nematodes (Fig. 4c). The amino acid substitutions were located in a cytosolic region of the meckelin protein immediately adjacent to a transmembrane domain based on current models of predicted meckelin protein structure^32^ (Fig. 4c).

### Ovine *TMEM67* transcripts containing the I681N and I687S amino acid substitutions were functionally impaired

**Figure 5:**
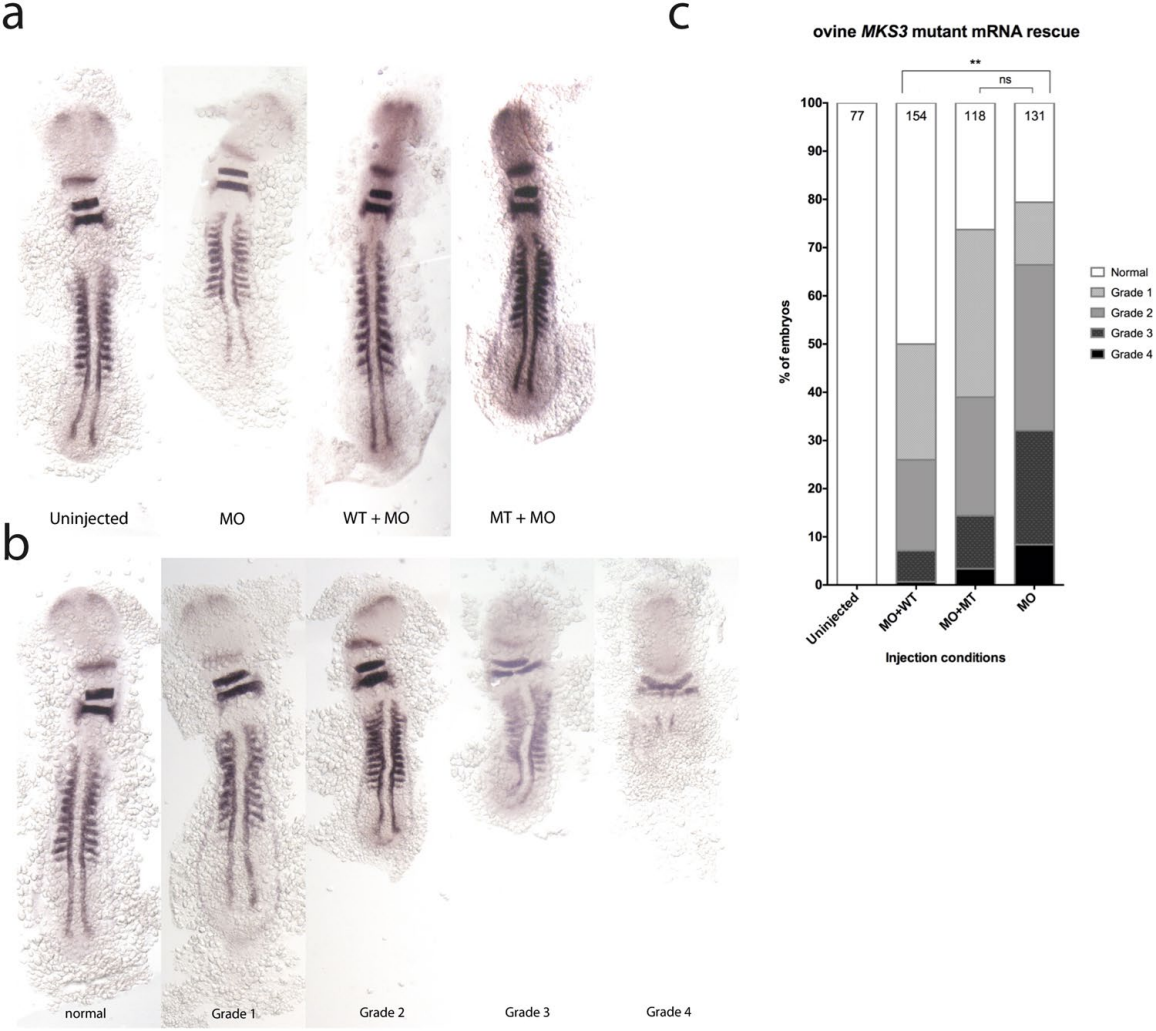
Loss of function of the *TMEM67* transcripts containing missense mutations in a zebrafish phenotype rescue assay. (**a**) Bright-field microscopy of morphant zebrafish embryos stained with riboprobes against *myod*, *pax2*, and *krox20* using *in situ* hybridization. Uninjected, uninjected embryos. MO, embryos injected with morpholino. WT + MO, embryos co-injected with *OaMKS3* mRNA plus MO. MT + MO embryos co-injected with mutant *OaMKS3*(*I681N*;*I687S*) mRNA plus MO. (**b**) Zebrafish embryos at the 11–12-somite stage stained by *in situ* hybridization with riboprobes against *myod*, *pax2*, and *krox20*. The graded embryos were injected with 0.17–0.33 pmol *mks3* MO, while normal embryos were injected with the solvent (Danieau buffer and phenol red dye mixture). The grade 1 morphant shows a shortened body axis, kinked notochords, and wide somites compared with the normal embryo, and exhibited one or two out of the three developmental aberrations (see Methods). Embryos with grade 2 phenotypes showed all three structural defects to a moderate degree. Grade 3 embryos showed more severe developmental aberrations. The embryos with grade 4 phenotype showed severe developmental malformations and died within or soon after 14 hpf. (**c**) Graph showing the phenotypic rescue of *mks3* morphant embryos co-injected at the 11–12 somite stage of development with ovine *OaMKS3* mRNA plus MO and scored using *in situ* hybridization (ISH). The percentages of the 11–12 somite rescued zebrafish embryos (with 50 ng mRNA) are shown in different morphological grades. Embryos at the 11–12-somite stage were assessed by *myoD*, *pax2*, and *krox20* labeling using ISH and observed under a bright-field microscope. Co-injection of MO plus 50 ng ovine wild type *OaMKS3* mRNA (MO + WT) resulted in a significant restoration of the normal phenotype in the morphant embryos compared with MO-injected embryos (**p < 0.0001, 2-tailed Fisher Exact test), whereas the co-injection of MO plus ovine mutant *OaMKS3*(*I681N*;*I687S*) mRNA (MO + MT) did not significantly restore the normal phenotype in the morphant embryos compared with morpholino-injected embryos (p = 0.2903). The number indicated at the top of each bar denotes the total number of embryos per injection condition. Abbreviations: MO, morpholino; WT, *OaMKS3* mRNA; MT, *OaMKS3*(*I681N*;*I687S*) mRNA; ns, not significant.

To determine whether the amino acid substitutions in ovine *TMEM67* (I681N and I687S) result in loss of meckelin function we carried out a zebrafish phenotype rescue assay at two different stages of zebrafish development, based on previous studies^30, 33^. Ovine meckelin protein has ~89.5% amino acid sequence identity to human meckelin (Supplementary Figure S2). Therefore we reasoned that, like human *TMEM67*, ovine *TMEM67* mRNA should also be able to phenotypically rescue the *tmem67* morphant phenotype. A splice-site disrupting morpholino oligonucleotide^33^ (MO) targeting zebrafish *tmem67* was used to disrupt meckelin function in zebrafish embryos from the 1-cell stage. Microinjection of the *tmem67* MO into zebrafish embryos resulted in a range of early phenotypes (see Fig. 5) caused by inhibition of splicing of endogenous meckelin mRNA in zebrafish embryos (Supplementary Figure S3). By mid-somitogenesis at 11–12 somites, which is equivalent to 14 hours post-fertilisation (hpf), meckelin disruption resulted in a shortened anterior-posterior axis, as previously described^30^. The meckelin disruption also generated a range of additional phenotypic abnormalities (Fig. 5a), including notochord, cardiac, tail, brain and otic placode defects. The morpholino-induced (morphant) phenotype was able to be rescued by co-injection of wild type human *TMEM67* mRNA^30, 33^, which was observed upon whole-mount analysis of *pax2*, *krox20* and *myod* expression patterns^30^ (Fig. 5b), and could also be observed by direct morphological analysis of zebrafish embryos (Supplementary Figures S4 and S5). Ovine wild-type *TMEM67* mRNA (*OakMKS3* mRNA) significantly rescued the morphant phenotype in *tmem67*-depleted embryos (p < 0.0001, Fig. 5c). In contrast, the microinjection of mutant ovine *TMEM67* mRNA containing both the I681N and I687S mutations (*OakMKS3*(*I1681N*;*I1687S* mRNA)) led to an impaired rescue of the morphant phenotype, and was not significantly different from those injected with MO alone (p = 0.2903, Fig. 5c). The results from the zebrafish phenotypic rescue assays were similar, irrespective of whether embryos were scored using *pax2*, *krox20* and *myod* whole-mount *in situ* hybridization (ISH) (in Fig. 5c), by direct observation of embryos at 14 hpf (Supplementary Figure S4), or by direct observation when the assay was carried out using 60–72 hpf embryos (Supplementary Figure S5). Taken together, these data suggest that the homozygous I681N and I687S amino acid mutations caused a significant loss of meckelin function *in vivo*.

### Meckelin was expressed in cyst epithelial cells and primary cilia, which were frequently detached from epithelial cells in affected lamb kidneys

**Figure 6:**
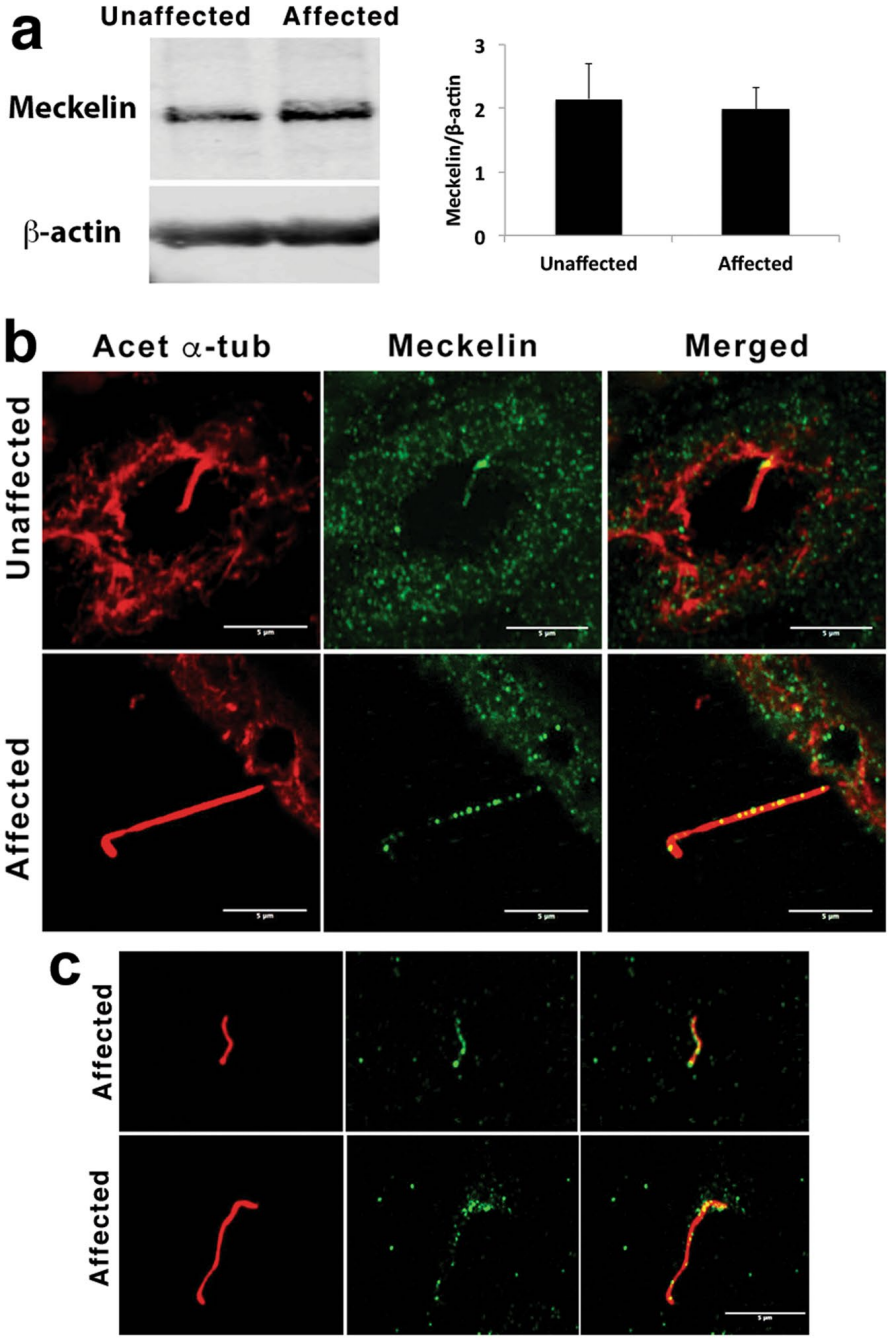
Meckelin expression in unaffected and affected lamb kidney epithelial cells and primary cilia. (**a**, left) Immunoblot showing expression of meckelin (~100 KDa) in cultured epithelial cells from an unaffected lamb kidney, and an affected lamb kidney. β-actin indicates equivalent protein loading in each lane. (**a**, right) Quantification of the meckelin band in the immunoblot, normalised to the loading control, shows no significant difference in relative protein expression. (**b**) Confocal maximum projection of acetylated α-tubulin (red) and meckelin (green) labelling in sections from unaffected and affected kidneys. In unaffected kidneys, meckelin was present within the cytoplasm of the epithelial cells and strongly localised to the base of the cilium. In contrast, meckelin was distributed along the length of the ciliary axonemes of affected cyst-lining cells. Meckelin distribution within the cytoplasm of cyst lining cells was similar to observations in unaffected epithelia (**c**) Acetylated α-tubulin (red) and meckelin (green) labelling on putative ciliary fragments located in the cyst lumen of affected kidneys (yellow indicates co-localisation in the merged image). Numerous smaller particles contained in the cyst luminal contents were also labeled with meckelin, but were not labeled with acetylated α-tubulin. Scale bars = 5 μm.

Meckelin expression in epithelial cells, which were cultured from affected and unaffected lamb kidneys, was analyzed by immunoblotting with an anti-meckelin antibody. An ~100 KDa protein was detected (the predicted molecular mass of meckelin variant 1 is 112 KDa, and variant 2 is 103 KDa) in both unaffected and affected lamb kidney epithelial cells (Fig. 6a). Western blot quantification revealed no significant difference in levels of meckelin protein between the affected and unaffected (normal) lamb kidney cells (p = 0.8), suggesting that there was no appreciable change in meckelin expression in affected lamb kidney cells as a result of the amino acid substitutions.

In normal kidneys, immunofluorescent detection of acetylated α-tubulin and meckelin suggested these proteins were co-expressed within epithelial cells of kidney tubules (Supplementary Figure S6). Analysis of meckelin expression in normal primary cilia revealed punctate expression concentrated at the base of the axoneme, which was co-localised with acetylated α-tubulin, with weaker punctate meckelin expression along the axoneme length (Fig. 6b).

In affected kidneys only intact cysts were examined to ensure that meckelin expression in all ciliary elements was detected, including ciliary elements contained in the contents of the cyst lumens. At high magnification punctate expression of meckelin was observed in association with epithelial cells in both affected and unaffected kidneys (Fig. 6b). Immunofluorescent co-localization of acetylated α-tubulin and meckelin was observed in the primary cilia of kidney cyst epithelial cells, with punctate expression of meckelin spread along the length of elongated cilia in a dispersed pattern (Fig. 6b). Punctate expression of meckelin was observed along presumed fragments of primary cilia that were located well away from the cyst epithelial lining. Numerous smaller meckelin-positive particulate bodies were also observed in the cyst luminal contents (Fig. 6c), that did not co-localise with acetylated α-tubulin. These meckelin-positive particles were not present in the cyst lumens of negative control sections (Fig. 6c).

### Renal primary cilia display morphological and length control defects

**Figure 7:**
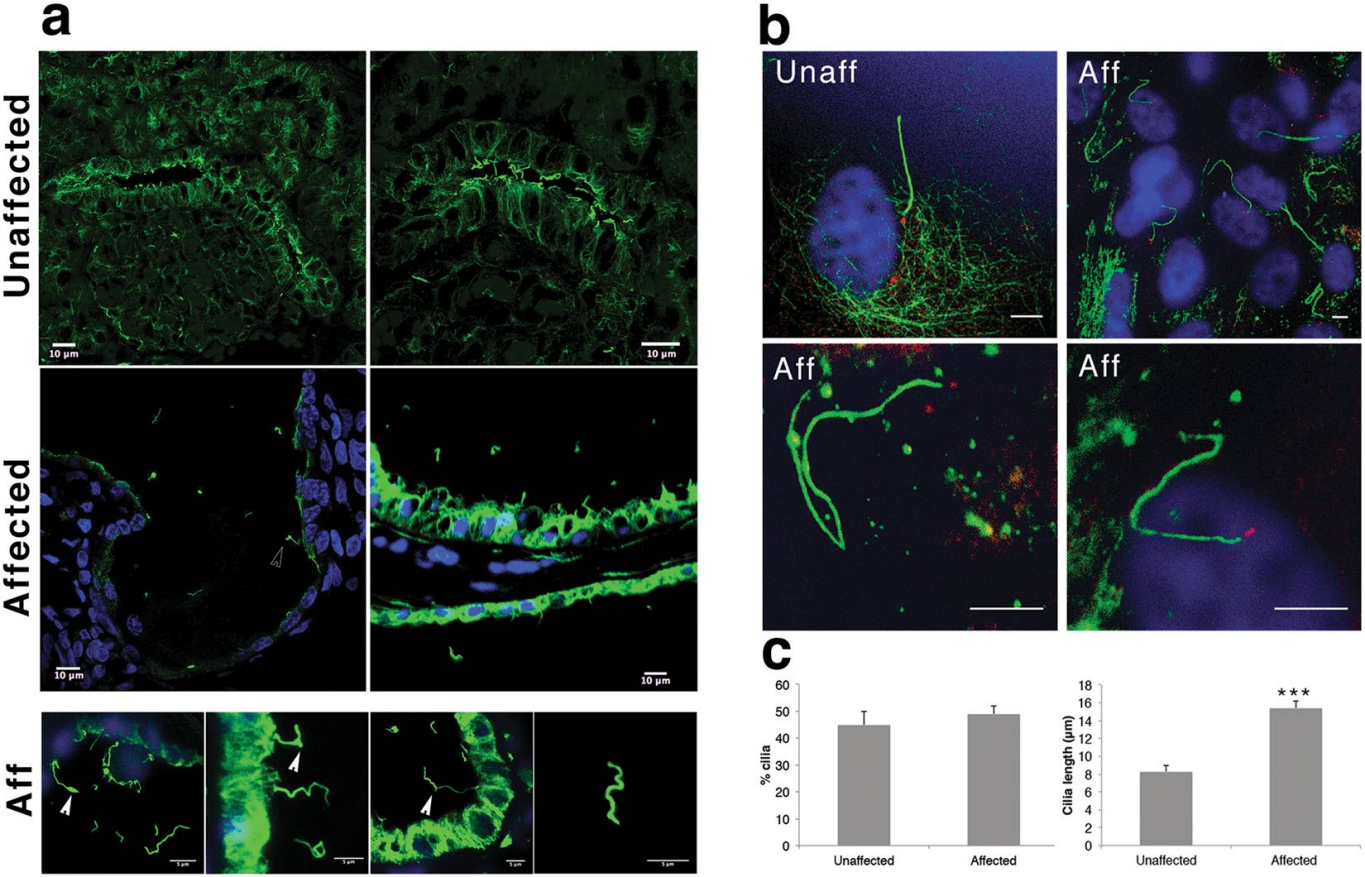
Defects in cilia morphology and length in affected lamb kidneys. (**a**) Acetylated α-tubulin labeling of primary cilia (green) in kidneys from unaffected and affected newborn lambs. Bottom panel (Aff) shows higher magnification images of primary cilia from affected lambs, which displayed a range of abnormal morphologies including bulbous projections along axonemes, sharp kinks and a wide range of lengths. Aceylated α-tubulin-positive structures were also present in the cyst luminal contents. The cell nuclei were stained with DAPI (blue). (**b**) Immunocytofluorescence labeling of primary cilia (acetylated α-tubulin; green) and centrioles (gamma-tubulin; red) in renal interstitial fibroblasts cultured from unaffected (Unaff) newborn lamb kidney, or affected (Aff) newborn lamb kidneys. In unaffected cells, acetylated α-tubulin was present in primary cilia, and gamma-tubulin localised in the two centrioles at the ciliary base. In affected fibroblasts, primary cilia were significantly longer that unaffected cilia and showed similar dymorphologies as observed *in situ*, including evidence of acute bending, bulbous projections along the axoneme and variably stained centrioles. Cell nuclei are stained with DAPI (blue). (**c**) Graphs of cilia incidence and cilia length measured in renal interstitial fibroblasts cultured from unaffected and affected newborn lamb kidneys (n ≥ 50 cilia per condition, from a total of 31 fields of view). ***p < 0.001. Scale bar = 10 µm.

In unaffected kidneys, primary cilia, labeled with acetylated α-tubulin (and in some cases arl13b), were present on tubular epithelial cells and cells within the interstitium (Fig. 7a). In affected kidneys, cilia were dysmorphic, and exhibited a wide range of lengths from very short to very long. Some cilia exhibited bulbous projections at the tip, and along the length of some of the axonemes were areas of weaker tubulin-positive labeling (Fig. 7a). In addition, acetylated α-tubulin-positive fragments, presumed to be detached epithelial cell cilia, were present in the cyst luminal contents at a considerable distance from the cyst wall (Fig. 7a; also see Supplementary Figure S7, and Supplementary movie M1). Confocal imaging through a 9 μm thick section showed that tubulin-positive fragments were not associated with cyst-lining cells, or with cells that were occasionally located within the cyst lumen (see Supplementary Movie M1). Double labeling for acetylated α-tubulin and ciliary GTPase Arl13b showed that fragments within the cyst lumen were most likely ciliary structures (Supplementary Figure S7), thus suggesting that these fragments of cilia had broken away from the apical surface of the cyst lining epithelial cells.

**Figure 8:**
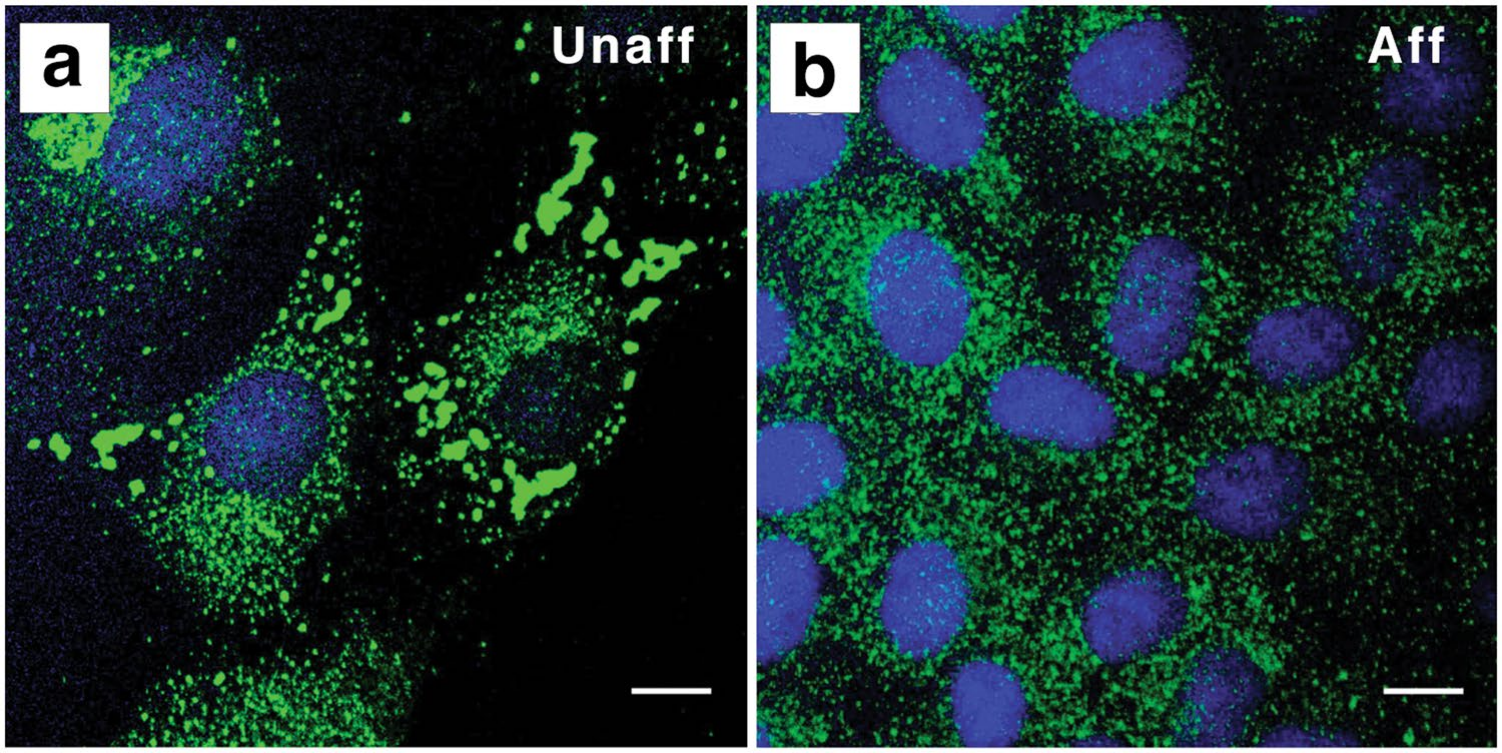
Distribution of 58 K Golgi-associated protein in affected and unaffected renal interstitial fibroblasts. (**a**,**b**) Immunofluorescence (green) labeling, using anti-Golgi 58 K protein antibody, was carried out in cultured renal interstitial fibroblasts cultured from unaffected (**a**) and affected (**b**) newborn lamb kidneys (**a**). The 58 K protein immunofluorescence was more evenly distributed in small particles in the cytoplasm of the affected renal interstitial fibroblasts, compared to the distribution in large and small particles of unaffected renal interstitial fibroblasts. Cell nuclei were stained with DAPI (blue). Scale bars = 10 μm.

To examine the cilia phenotype further, we cultured interstitial fibroblasts from unaffected lamb kidneys and found that unaffected cells exhibited relatively straight primary cilia (Fig. 7b). In contrast, primary cilia of cultured interstitial fibroblasts from an affected lamb kidney exhibited an array of dysmorphologies, including kinks and twists along the length of the axoneme, acute bending, vesicular-like bodies contained in the axoneme, and basal centrioles that were variably detected with gamma tubulin (Fig. 7b). Unaffected kidney interstitial fibroblasts exhibited a similar incidence of cilia (45% ± 7%) to that of affected interstitial fibroblasts from two different animals (49% ± 3%; Fig. 7b). However, affected kidney interstitial fibroblasts from two different animals had significantly longer cilia (15.1 ± 1.8 µm) compared to interstitial fibroblasts from an unaffected kidney (8.3 ± 1.0 µm; Fig. 7b, p < 0.01). Additionally, since recent studies have suggested a potential role for stable Golgi-cilia associations in the spatial control of ciliogenesis^34^, we also investigated the 58 K Golgi-associated protein. Using immunofluorescence localization studies, we found that this protein was more evenly distributed amongst smaller particles in the cytoplasm of affected kidney interstitial fibroblasts than in unaffected cells (Fig. 8).

## Discussion

Here we describe a hepatorenal fibrocystic syndrome in sheep, associated with deleterious amino acid substitutions in exon 20 of *TMEM67*, identified by homozygosity mapping in affected lambs. The genotypic and phenotypic features were identified in two distantly related flocks of New Zealand sheep breeds (Coopworth and Perendale)^31^, exhibiting an essentially identical disorder. The two diseased flocks (flocks A and B, respectively) were identified independently on NZ farms located over 500 km from each other, and no interbreeding between the two flocks has occurred over the last 40 years. The diseased lambs in both flocks A and B exhibited an indistinguishable perinatal lethal recessive syndrome with multi-organ involvement.

Several observations support the notion that the amino acid substitutions in *TMEM67* cause the phenotype in the affected lambs. These include: (i) the missense mutations in *TMEM67* were identified in a large region of homozygosity, which was shared in affected lambs; (ii) sequencing of 55 predicted genes in the chromosome 9 and 11 regions of shared homozygosity, followed by analysis of the exons against reference sequences identified only two missense variants that matched expected inheritance and phenotypic characteristics associated with the variants; (iii) homozygosity of the missense mutations in *TMEM67* was identified concordantly with affected status of the offspring in both flocks; (iv) the two amino acid substitutions resulted in loss of function of *TMEM67*, as determined in zebrafish phenotype rescue assays; (v) the missense mutations in the *TMEM67* gene on ovine chromosome 9 are a plausible candidate for the cause of the disorder, since mutations in this gene have been identified in human patients with the inherited lethal recessive PKD disorder, MKS3^9^, which is associated with a number of features similar to those observed in the affected lambs, as discussed below.

The observation that affected lambs exhibited a fetal mid-gestational onset of PKD with liver fibrosis and a ductal plate malformation, together with perinatal lethality and mutations in *TMEM67*, suggests that the syndrome in the affected lambs is a model for the human ciliopathy, MKS3. MKS3 is a hepatorenal fibrocystic syndrome in which ductal plate malformation has been reported^28^. The phenotypic heterogeneity of MKS3 abnormalities observed in patients with *TMEM67* mutations has significant overlaps at each end of its spectrum with two other ciliopathies–Joubert syndrome and nephronophthisis. Thus some patients with mutations in *TMEM67* have been diagnosed with Joubert syndrome (JBT6) or with nephronophthisis (NPHP11). However, since neither Joubert syndrome nor nephronopthisis patients exhibit both liver fibrosis and kidney cysts together in the same patient, they are not considered to be hepatorenal fibrocystic syndromes. Unlike MKS3 in humans^23^, the affected lambs in this study uniformly lacked polydactyly, as well as overt developmental brain abnormalities, including encephalocoele. In addition, other brain abnormalities, such as those commonly observed in the rodent models of MKS3, for example exencephaly, meningocoele and hydrocephalus in the rat (*Wpk*) and mouse (eg *bpck*) models of MKS3^7, 9, 26–29^, were absent in the affected lambs. Therefore, it is possible that the phenotype in the affected lambs has a greater similarity to the phenotype observed in NPHP11 nephronophthisis patients, who have mutations in *TMEM67*, but lack occipital encephalocoele.

Loss of function of the protein encoded by *TMEM67* as a result of the two ovine missense variants was characterized using a zebrafish phenotype rescue assay. *TMEM67* mRNA transcripts containing the two missense mutations rescued a *tmem67* knockdown (“morphant”) phenotype in zebrafish much less efficiently than the wild type ovine *TMEM67* mRNA transcripts. While it remains possible that just one of the two mutations might be sufficient to confer the burden of pathogenicity, we have so far only tested the pathogenicity of both mutations simultaneously, and these data were consistent with the notion that both missense variants in *TMEM67* together confer reduced function to the meckelin protein and are pathogenic. Previously it has been suggested that hypomorphic *TMEM67* mutations are associated with patients who have no neurological signs^35^. We therefore speculate that the ovine mutations could be associated with a hypomorphic phenotype, which might then explain the observation that affected lambs in flocks A and B lacked occipital encephalocoele, a feature that is generally considered to be a cardinal sign of Meckel syndrome.

The missense variants identified in ovine *TMEM67* were non-conservative amino acid changes (p.[(Ile681Asn; Ile687Ser)]) occurring within a conserved region, which is part of an intracellular cytoplasmic loop of the meckelin protein^8^. Although the presence of two missense mutations occurring *in cis* in the same exon is relatively uncommon, several pairs of mutations have been reported within the same exon of recessive genes in other diseases, such as in profound biotinidase deficiency^36^, cystic fibrosis^37^, aspartylglucosaminuria^38^, and Gaucher disease^39^. In the cytosolic region (aa ~500–590) of the meckelin protein, which is near the site of the sequence variants identified in this study, there have so far been only six missense mutations and an exon skipping deletion identified in patients. The six mutations have been associated with a wide range of disease phenotypes, including Joubert syndrome, another syndrome (COACH syndrome – consisting of cerebellar vermis hypo/aplasia, oligophrenia, congenital ataxia, ocular coloboma, and hepatic fibrosis), MKS, and ARPKD-like syndromes^32^. Neither of the amino acid substitutions identified in the affected sheep has previously been reported in the literature in human *TMEM67*, or in any other species.

Meckelin has been shown to physically interact with several proteins^8, 33^, including proteins mutated in disorders such as nephronophthisis and Joubert syndrome^40^. Meckelin is thought to play a role in the genesis or maintenance of the transition fibres that secure the distal end of the ciliary basal body to the ciliary membrane junction, and also in the Y-shaped linkers of the transition zone that tether the proximal axonemal microtubules to the ciliary membrane immediately distal to the basal body^41^. While primary cilia with normal appearance were present on all epithelial cells in unaffected sheep kidneys, in contrast the primary cilia in kidney cysts were of varying lengths (some of the cilia contributing to Fig. 7d were up to 30 μm). Multiciliated cells were also observed in cyst epithelial cells from affected lambs (data not shown). Defects in the cilia length, and the formation of multi-ciliated cells appear to be common features associated with abnormalities in transitional zone complex proteins. Length control defects of primary cilia have been reported in mouse and rat models containing mutations in *TMEM67* ^7, 42^.

In addition to cilia dysmorpholgies, a large number of primary cilia fragments, which were presumably broken off from renal cystic epithelial cells, were present in the cyst luminal contents. During tissue processing, whole intact cysts were fixed and sectioned. The fixed cyst luminal contents were transferred along with adjacent tissue onto the glass slides, as we typically observed debris contained in the lumen of cysts. As is the case with the intact tissue, we presume cyst lumen contents form a stable cohesive interaction with the surface of the glass, and as a result the cilia fragments were retained during the subsequent incubation and washing steps. These cilia fragments appeared to be a consistent and defining characteristic of the ciliary phenotype. Imaging studies (see Supplementary Movie M1) showed that the cilia were not attached to cells located above or below the focal plane, and fragments were observed a considerable distance from the cyst lining cells within the luminal contents of very large cysts. The cause of this finding remains unknown, although no ciliary fragments were observed in unaffected tubules. One possible interpretation of this is that primary cilia on cyst epithelial cells of affected kidneys are exposed to greater hydrostatic pressures within an expanding cyst, which may result in mechanical breakage of the extremely long cilia. This may explain the variable cilia length observed within cysts, and could suggest that abnormally short cilia were a result of cilia fracturing. However, it is acknowledged that, while both unaffected and affected kidneys were fixed and processed identically, further work is required to determine if the ciliary fragmentation is present *in vivo*.

When examining cultured renal interstitial fibroblasts from affected lambs, the ciliary phenotype was characteristic of the abnormally long primary cilia observed in cyst cells *in situ*. These cilia were also similarly distorted with multiple intra-ciliary vesicle-like bodies, compared to renal interstitial fibroblasts from unaffected lamb kidneys. In addition, we found that the 58 K Golgi-associated protein was more evenly distributed amongst smaller particles in the cytoplasm of affected kidney interstitial fibroblasts compared to unaffected cells, which is potentially interesting in light of recent studies implicating stable Golgi-cilia associations in the spatial control of ciliogenesis^34^. However, in further immunofluorescence work using additional Golgi markers, including Golgin97 and WGA, we did not observe equivalent differences in the Golgi organization between affected and unaffected kidney interstitial fibroblasts (data not shown). The implications of these findings are presently unclear, and further studies will be required to determine the significance of the differences in 58 K protein localization in affected and unaffected cells. Nevertheless, the fact that morphological abnormalities of primary cilia were observed in affected kidneys suggests that the meckelin amino acid substitutions may have had an impact on the biogenesis or the maintenance of the cilia. To our knowledge, no similar description of severely dysmorphic primary cilia has been reported previously in association with *TMEM67* mutations.

In conclusion, we describe a hepatorenal fibrocystic syndrome in sheep associated with pathogenic amino acid substitutions in exon 20 of ovine *TMEM67*. The kidney and liver abnormalities in the affected lambs were similar to those observed in the Meckel/Joubert/Nephronophthisis constellation of abnormalities in humans. These observations expand our knowledge of the phenotypic spectrum of ciliopathies related to the Meckel/Joubert/Nephronophthisis constellation and describe a sheep flock that constitutes the first large animal model of this group of ciliopathies. Dysmorphic primary cilia, and evidence of their fragility were also observed in the renal cystic epithelial cells and cultured renal interstitial fibroblasts of affected lambs; these observations expand the pivotal role that meckelin plays in primary cilia function. Further investigations using large animal models, such as the model used in these studies, will allow greater understanding of ciliopathies, and potentially could be used in the development of novel therapeutic strategies to treat ciliopathies, such as Meckel syndrome, Joubert syndrome, or Nephronophthisis.

## Materials and Methods

### Ethics statement

All experimental procedures were performed in accordance with relevant guidelines and regulations with respect to international principles of laboratory animal care and were approved by the University of Otago Animal Ethics Committee and Massey University Animal Ethics Committee (protocol numbers 09/24 and 09/111).

### Animals

Sheep flock A^31^ is a Perendale breed sourced from a Canterbury (New Zealand) farm. Sheep flock B^31^ is a Coopworth breed sourced from a Southland (New Zealand) farm. Affected lambs were produced by backcrossing carrier female offspring with a carrier ram, but rams from flock A were never bred with flock B, and *vice versa*. Pregnant ewes were ultrasound scanned during pregnancy to identify affected fetuses with polycystic kidneys. Affected lambs were immediately euthanized following birth with an overdose of intravenous pentabarbitol. Zebrafish were raised and maintained in the Otago Zebrafish Facility (OZF) at the Department of Pathology, University of Otago. The wild type AB zebrafish strain was used in this study.

### Tissue collection/processing, and tissue culture for primary cilia preparations

One dissected kidney was used for isolation of primary cell cultures, and the other kidney used for RNA and DNA isolation and preparing tissue sections using standard protocols. For histology and immunofluorescence tissue sections were cut at 5–10 µm. Hematoxylin and Eosin (H&E) and Masson’s trichrome staining were carried out as per the manufacturer’s guidelines (Sigma-Aldrich, St Louis, MO, USA). Explant tissue cultures were established for renal interstitial fibroblasts, and renal epithelial cells, as previously described^43^. Cells (1 × 10^7^) were seeded onto clean, sterile glass coverslips (*In Vitro* Technologies, Rahway, NJ, USA) in 24 well tissue culture plates (Corning, New York, USA) in advanced D-MEM + 10% fetal bovine serum (FBS) (Gibco, Thermo Fisher Scientific, Bartlesville, OK, USA). Once cells reached 90% confluence, FBS was removed to promote primary cilia expression. Serum-free media was removed after 3 days and replaced with freshly prepared 4% paraformaldehyde in phosphate buffered saline (PBS) (Sigma-Aldrich, St Louis, USA) for 1 hr at 4 °C for immunocytochemistry. Coverslips for immunocytochemistry were washed extensively with PBS and stored at 4 °C in PBS + 0.1% sodium azide.

### Immunoblotting

Immunoblotting was performed as previously described^44^ with the following modifications: Briefly, cell lysates were prepared in radioimmunoprecipitation assay (RIPA) buffer, and quantitated with the bicinchoninic acid (BCA) assay. Protein lysates (30 μg) were electrophoresed and transferred. For primary antibodies, a 1/2000 dilution of meckelin antibody (product number GTX110171, GenTex, Irvine, CA, USA), or a 1/10,000 dilution of β-actin antibody (Sigma-Aldrich, St Louis, MO, USA), were used. For secondary antibodies, 1/15,000 dilutions of goat anti-rabbit IRDye 800 CW for meckelin (LiCor, Lincoln, NE, USA), and goat anti-mouse IRDye 680RD for actin (LiCor, Lincoln, NE, USA) were used. Blocking was carried out with 10% low fat milk/TBS for 1 h, and primary antibodies were incubated in 2.5% FBS/TBST/0.01% azide overnight at 4 °C. Secondary antibody incubations were carried out in 5% LFM/TBST, and washes were carried out with TBST.

### Immunofluorescence

Immunofluorescence was carried out using standard procedures, briefly described as follows: The following primary antibodies were used: monoclonal anti-acetylated tubulin clone 6–11-B1, 1:1250 (Sigma-Aldrich, St Louis, MO, USA), C3B9, 1:10^45^, monoclonal anti-gamma-tubulin, 1:1000 (Sigma-Aldrich, St Louis, MO, USA), rabbit polyclonal anti-meckelin, 1: 250 (product number NBP1-06590, Novus, St Charles, MO, USA), monoclonal anti-golgi 58K protein, 1:1000 (Sigma-Aldrich, St Louis, MO, USA), monoclonal anti-human smooth muscle actin, 1:1000 (Dako, Carpinteria, CA, USA), rabbit polyclonal anti-arl13b (1:100; Proteintech, Rosemount, IL USA). Secondary antibodies used included: Alexa Fluor 488 goat anti-mouse IgG, Alexa Fluor 546 goat anti-mouse IgG, and Alexa Fluor 488 donkey anti-rabbit IgG (all preceding antibodies were from Molecular Probes, Thermo Fisher Scientific, Bartlesville, OK, USA). Sections (10 μm thick) underwent antigen retrieval by incubating the slides in 0.1 M citrate buffer for 5 min at 100 °C. Antigen retrieved wax sections on slides, and cell monolayers on coverslips, were washed extensively in PBS before permeabilisation in 0.5% Triton-X in PBS, 0.1% BSA. Brightfield and epifluorescence using a 63x or 100x oil immersion lens on an Olympus microscope (AX70) and Zeiss 510 Confocal Laser Scanning Microscope were carried out in the Otago Centre for Confocal Microscopy, and the Biomedical Imaging Research Unit, The University of Auckland.

### SNP genotyping

DNA was extracted from ear, kidney or liver tissue of affected and unaffected lambs using a Promega Wizard DNA isolation kit (Promega Corporation, Fitchburg, WI, USA) according to the manufacturer’s instructions. The amount and purity of DNA was assessed using a Nanodrop spectrophotometer (Thermo Fisher Scientific, Bartlesville, USA). The genotyping of 54,241 evenly distributed polymorphic SNP markers across the genome was performed using the Illumina Ovine SNP50 BeadChip at Illumina Inc. (San Diego, CA, USA). A standard PCR and ligation-free protocol was used, which routinely achieves high average call rates and high accuracy.

### Genome-wide homozygosity mapping

From SNP genotyping, consecutive SNPs exhibiting homozygosity-by-descent (identical by descent or IBD regions) were analysed as previously described^46, 47^. An in-house script was used to identify common regions of consecutive homozygous SNP loci across affected animals. The same procedure was applied to a known carrier to ensure that the identified region was not simply fixed across all animals. A threshold of 8 SNPs was used to define a candidate consecutive homozygous region after examining IBD fragment sizes for different threshold numbers of SNPs for 14 affected lambs of flock B and 6 affected lambs of flock A. To accommodate occasional genotyping/phenotyping errors or map errors in Ovine Genome Assembly v1.0, a sliding window approach was used to report the percentage of consecutive SNPs that were homozygous and concordant across all sheep to identify regions of enriched homozygosity. Candidate genes in IBD regions were examined by comparing homology between bovine and ovine (Ovine Genome Assembly OARv1.0; http://genome.ucsc.edu/cgi-bin/hgTracks?db=oviAri1) genomes.

### Genome resequencing and candidate gene sequencing

Whole genome sequencing was carried out by New Zealand Genomics Ltd (NZGL, Dunedin, NZ), with preparation of TruSeq DNA libraries with an average insert size of 420 bp from genomic DNA of an affected lamb with standard protocols. Next Generation Sequencing (NGS) of the ovine DNA sample was carried out using the Illumina HiSeq 2000 platform with v1.5 chemistry reagents and flowcells (NZGL, Dunedin, NZ). Each sequencing run produced 83–85% > Q30 2 × 100 bp paired-end reads, with an average of >30-fold genomic coverage, and an average insert size of 420 bp. Sequence reads were initially mapped to the ovine genome assembly OARv1.0, and later to genome assembly OARv3.1. Mapping parameters were optimised for the sheep genome.

To sequence exon 20 of *TMEM67*, PCR primers flanking the coding regions of ovine *TMEM67* were designed (Supplementary Table S8). Sanger sequencing of PCR products was carried out by the Massey Genome Service, (Massey University, Palmerston North, NZ) as described previously^48^ on an ABI3730 DNA Analyzer using BigDye® Terminator v3.1 chemistry and cycle sequencing (Applied Biosystems Inc. Foster City, CA, USA), and sequences aligned using Geneious (Biomatters Ltd., Auckland, NZ) to the bovine and human *TMEM67* reference mRNA sequences (Genbank: NM_001205299.1; Genbank: NM_153704.5). Structure/function predictions were carried out using A-GVGD and SIFT software, and the Polyphen programme^49^, using sequences from sheep, rat (ID: NP_00101386.2), mouse (ID: NP_808529), cow (ID: XP_583257), human (ID: AAH32835), zebrafish (ID: XM_695882.4), and chicken (ID: XM_418334.2).

### Restriction enzyme digestion (PCR-restriction fragment length polymorphism (RFLP) genotyping)

Genotyping was carried out following amplification of exon 20 of *TMEM67* using PCR primers (refer to Supplementary Table S8) for 30 cycles at 95 °C for 30 sec, 55 °C for 30 sec, 72 °C for 1 min, followed by digestion of the PCR product (612 bp) using the restriction enzyme *Apo*I (New England Biolabs Inc., Ipswich, MA, USA) at 50 °C. The products were analyzed on a 2.0% (w/v) Nusieve agarose gel (Lonza, Allendale, NJ, USA), giving bands sizes of 140 bp, 208 bp, 264 bp for wild type, 140 bp and 472 bp for homozygous mutant, and 140 bp, 208 bp, 264 bp, and 472 bp for heterozygous mutation carriers.

### mRNA and morpholino (MO) injections of zebrafish embryos

An anti-sense *tmem67* splice-blocking morpholino (MO) (GeneTools, Philomath, OR, USA) was used to target the *tmem67* intron 3/exon 3 boundary (5′-GTAAAAATGACAAGCGCCTACCCAG-3′). The MO oligonucleotide^33^ was injected at a concentration of 0.33 pmol/nL (~2.95 ng)/embryo. The use of this MO, including the selection of a suitable working concentration range was based on previous reports^30, 33, 50^. An RT-PCR assay was carried out (Supplementary Figure S3) on injected or uninjected zebrafish embryos to confirm that an altered splicing of zebrafish *tmem67* transcripts had occurred following anti-sense *tmem67* MO treatment, as previously described^33^, except that a pool of 20 embryos was used for each group. Briefly, 500 ng of RNA extracted from pooled zebrafish embryos at 72 hpf (using a Qiagen kit according the manusfacturer’s instructions) was reverse transcribed using a qScript cDNA SuperMix kit (Quanta Biosciences), followed by PCR using a FastStart Taq DNA Polymerase kit (Roche) for 35 cycles at 50 °C (30 sec), 72 °C (30 sec), and 94 °C (30 sec), and the PCR products were resolved on a 2% agarose gel. The RT-PCR primers used to detect altered splicing in zebrafish *tmem67* were *mks3*F: GAGCCTGTCTTGCGTGAAAT, and *mks3*R: CCGCAAACACAAGACTGAGA.

To generate TMEM67 mRNA the ovine wild type *TMEM67* and mutant *TMEM67* cDNAs were individually amplified using the Roche Expand Long Template PCR System 1 from full-length cDNA templates with PCR primers 5′-GTCTGCACCATGGCGACGG-3′ and 5′-TGTGTTGTGGATTTGGCTTG-3′ (Roche, Indianapolis, IN, USA). The cDNAs were subcloned into the pCR2.1-TOPO vector using an Invitrogen TOPO-TA Cloning® method (Invitrogen, Thermo Fisher Scientific, Bartlesville, OK, USA). The resulting plasmids were linearized with *Hind*III and *in vitro* transcribed with T7 RNA polymerase (Roche, Indianapolis, IN, USA) using the mMessage mMachine Kit (Ambion, Thermo Fisher Scientific, Bartlesville, OK, USA). Rescue experiments were performed with the co-injection of 0.33 pmol *tmem67* MO and 50 ng ovine *TMEM67* mRNA diluted in nuclease-free water into 1-cell stage embryos.

### Phenotypic analysis of zebrafish embryos

Developmental defects due to *tmem67* gene knockdown in 11–12 somite zebrafish (14 hpf) and 60–72 hpf zebrafish included shortened body axis, thin and wide somites, and distorted notochord^30^. The phenotypes were analyzed directly under brightfield microscopy or following staining by *in situ* hybridization with riboprobes detecting *pax2*, *krox20* and *myod* expression, and were categorized into 5 groups based on the severity of the structural abnormalities (refer to Fig. 5B): normal phenotype, grade 1 (embryos with one or two out of the three defects, or with three defects to a mild degree; mild proximal or distal bent notochord with mild cardiac oedema and/or tail abnormalities), grade 2 (embryos with three defects to a moderate degree; with grade 1 defects but greater in severity, plus shortened body axis), grade 3 (embryos with three defects to a severe degree; with grade 2 defects but greater in severity, plus severe body axis distortion and/or brain and otic placode anomalies), and grade 4 (embryos with severe developmental malformations and significant structure disruption).

### Photomicrography, *in situ* hybridization and statistical analysis of zebrafish embryos

The *in situ* hybridization of zebrafish embryos using *myoD*, *pax2*, and *krox20* riboprobes was performed using standard protocols^51^. *MyoD*, *pax2*, and *krox20* riboprobes were synthesized using T7, SP6, and T3 RNA polymerase (Roche, Indianapolis, IN, USA) respectively, from *BamHI*-, *KpnI-*, and *PstI-*digested cDNAs. For direct brightfield observations, zebrafish embryos were placed in methylcellulose on a depression slide, while for *in situ* hybridization staining, embryos were placed in 70% glycerol on a depression slide. Imaging of zebrafish embryos was performed with a Leica stereomicroscope. Statistical analyses of the phenotypic rescue experiments of zebrafish under different microinjection conditions were performed using a two-tailed Fisher Exact Test, where grade 1 to grade 4 embryos were combined together as being morphologically defective (morphant) embryos^30^. The numbers of morphant versus normal embryos co-injected with *tmem67* MO and mutant *MKS3* mRNA were compared to the numbers of morphant versus normal embryos in clutches injected with MO alone using Fisher Exact Test, and similarly the numbers of morphant versus normal embryos co-injected with *tmem67* MO and wild type ovine *MKS3* mRNA were compared to morphant versus normal embryos in clutches injected with MO alone.

## Electronic supplementary material

Supplementary material

Supplementary movie
