# Supplementary material for: An ovine hepatorenal fibrocystic model of a Meckel-like syndrome associated with dysmorphic primary cilia and TMEM67 mutations
Source: Sci Rep. 2017 May 9;7:1601. doi: 10.1038/s41598-017-01519-4 (PMC5431643; doi:10.1038/s41598-017-01519-4)
Supplement: Supplementary file 1 — Supplementary material [file 41598_2017_1519_MOESM1_ESM.pdf]

Supplementary Material for:

An ovine hepatorenal fibrocystic model of a Meckel-like syndrome associated with dysmorphic primary cilia and *TMEM67* mutations

C Stayner[§1], CA Poole[§2,6], SR McGlashan[3], M Pilanthananond[1], R Brauning[4], D Markie[1], B Lett[1], L Slobbe[1], A Chae[1], AC Johnstone[5], CG Jensen[3], JC McEwan[3], K Dittmer[5], K Parker[2], A Wiles[1], W Blackburne[1], A Leichter[1], M Leask[1], A Pinnapureddy[1], M Jennings[2], JA Horsfield[1], RJ Walker[2], MR Eccles[*1].

Corresponding author: Michael R. Eccles

Department of Pathology, Dunedin School of Medicine,

University of Otago, PO Box 913, Dunedin, New Zealand

Ph: +64 3 479 7878; Fax: +64 3 479 7136

michael.eccles@otago.ac.nz

Supplementary Tables

Supplementary Table S1. SNPs on chromosomes 9 and 11 contained in the regions of shared homozygosity in affected lambs.

SNP ID*	Ovine chrom-osome	Shared homozygous allele in flock A	Shared homozygous allele in flock B	Position on chromosome	
				v1.0	v3.1
OAR9_87942007.1	9	AA	TT	87942007	83056186
OAR9_88113366.1	9	GG	GG	88113366	83231298
OAR9_88126600.1	9	GG	GG	88126600	83243978
OAR9_88173540.1	9	AA	CC	88173540	83301647
s29382.1	9	GG	GG	88207064	83322916
s45417.1	9	AA	GG	88230849	N/A
s31334.1	9	AA	TT	88291244	83420640
s51965.1	9	AA	TT	88314648	83438870
s50770.1	9	AA	CC	88360997	83480450
OAR9_88387736.1	9	GG	AA	88387736	83506476
OAR9_88429424.1	9	GG	TT	88429424	83549788
s68264.1	9	AA	AA	88461946	83583396
s14982.1	9	GG	CC	88469132	83592801
OAR9_88521505.1	9	AA	TT	88521505	83638545
OAR9_88555527.1	9	CC	AA	88555527	83672030
OAR9_88619765.1	9	AA	AA	88619765	83737611
OAR9_88658895.1	9	CC	TT	88658895	83776909
OAR9_88702477.1	9	GG	CC	88702477	83817952
s58148.1	9	GG	AA	88749619	83874127
OAR9_88784528.1	9	CC	AA	88784528	83909454
OAR9_88826213.1	9	GG	TT	88826213	83946079
OAR9_88856945_X.1	9	AA	CC	88856946	83973853
OAR9_88886896.1	9	AA	TT	88886896	83996100
OAR9_88922262.1	9	GG	CC	88922262	84027999
OAR9_88974726.1	9	AA	AA	88974726	84093296
OAR9_89018996.1	9	AA	AA	89018996	84133462
s56248.1	11	TT	TT	27807356	26571629
s31301.1	11	AA	AA	27857758	26623188
OAR11_27937828.1	11	CC	CC	27937828	26701950
s36839.1	11	GG	GG	28072278	26799241
s72626.1	11	CC	CC	28404422	26872280
OAR11_28473036.1	11	TT	TT	28473036	26939891
s49534.1	11			28713152	N/A
s49406.1	11	CC	CC	28950668	27285522

Legend for supplementary Table S1. * The unique identifier of each SNP, shared genotype and position on the sheep genome v1.0 as well as v3.1 is shown (N/A, the SNP was not present in v3.1).

Supplementary Table S2. Chromosome 9 SNP50 Beadchip SNP (genome assembly v1.0) genotypes of affected lambs of flocks A and B. In one affected lamb SNP genotypes were not concordantly homozygous for much of the region (red).

			Affected lamb number																			
SNP ID	Chr	Chr. Posit-ion	1	2	3	4	5	6	7	8	9	10	11	12	13	14	15	16	17	18	19	20
OAR9_87942007.1	9	87942007	A<br>A	A<br>A	A<br>A	A<br>A	A<br>A	A<br>A	T<br>T	T<br>T	T<br>T	T<br>T	T<br>T	T<br>T	T<br>T	T<br>T	T<br>T	T<br>T	T<br>T	T<br>T	T<br>T	T<br>T
OAR9_88113366.1	9	88113366	G<br>G	G<br>G	G<br>G	G<br>G	G<br>G	G<br>G	G<br>G	G<br>G	G<br>G	G<br>G	G<br>G	G<br>G	G<br>G	G<br>G	G<br>G	G<br>G	G<br>G	G<br>G	G<br>G	G<br>G
OAR9_88126600.1	9	88126600	G<br>G	G<br>G	G<br>G	G<br>G	G<br>G	G<br>G	G<br>G	G<br>G	G<br>G	G<br>G	A<br>G	G<br>G	G<br>G	G<br>G	G<br>G	G<br>G	G<br>G	G<br>G	G<br>G	G<br>G
OAR9_88173540.1	9	88173540	A<br>A	A<br>A	A<br>A	A<br>A	A<br>A	A<br>A	C<br>C	C<br>C	C<br>C	C<br>C	C<br>C	C<br>C	C<br>C	C<br>C	C<br>C	C<br>C	C<br>C	C<br>C	C<br>C	C<br>C
s29382.1	9	88207064	G<br>G	G<br>G	G<br>G	G<br>G	G<br>G	G<br>G	G<br>G	G<br>G	G<br>G	G<br>G	G<br>G	G<br>G	G<br>G	G<br>G	G<br>G	G<br>G	G<br>G	G<br>G	G<br>G	G<br>G
S45417.1	9	88230849	A<br>A	A<br>A	A<br>A	A<br>A	A<br>A	A<br>A	G<br>G	G<br>G	G<br>G	G<br>G	G<br>G	G<br>G	G<br>G	G<br>G	G<br>G	G<br>G	G<br>G	G<br>G	G<br>G	G<br>G
s31334.1	9	88291244	A<br>A	A<br>A	A<br>A	A<br>A	A<br>A	A<br>A	T<br>T	T<br>T	T<br>T	T<br>T	T<br>T	T<br>T	T<br>T	T<br>T	T<br>T	T<br>T	T<br>T	T<br>T	T<br>T	T<br>T
s51965.1	9	88314648	A<br>A	A<br>A	A<br>A	A<br>A	A<br>A	A<br>A	T<br>T	T<br>T	T<br>T	T<br>T	T<br>T	T<br>T	T<br>T	T<br>T	T<br>T	T<br>T	T<br>T	T<br>T	T<br>T	T<br>T
s50770.1	9	88360997	A<br>A	A<br>A	A<br>A	A<br>A	A<br>A	A<br>A	C<br>C	C<br>C	C<br>C	C<br>C	C<br>C	C<br>C	C<br>C	C<br>C	C<br>C	C<br>C	C<br>C	C<br>C	C<br>C	C<br>C
OAR9_88387736.1	9	88387736	G<br>G	G<br>G	G<br>G	G<br>G	G<br>G	G<br>G	A<br>A	A<br>A	A<br>A	A<br>A	A<br>G	A<br>A	A<br>A	A<br>A	A<br>A	A<br>A	A<br>A	A<br>A	A<br>A	A<br>A
OAR9_88429424.1	9	88429424	G<br>G	G<br>G	G<br>G	G<br>G	G<br>G	G<br>G	T<br>T	T<br>T	T<br>T	T<br>T	T<br>T	T<br>T	T<br>T	T<br>T	T<br>T	T<br>T	T<br>T	T<br>T	T<br>T	T<br>T
s68264.1	9	88461946	A<br>A	A<br>A	A<br>A	A<br>A	A<br>A	A<br>A	A<br>A	A<br>A	A<br>A	A<br>A	A<br>A	A<br>A	A<br>A	A<br>A	A<br>A	A<br>A	A<br>A	A<br>A	A<br>A	A<br>A
s14982.1	9	88469132	G<br>G	G<br>G	G<br>G	G<br>G	G<br>G	G<br>G	C<br>C	C<br>C	C<br>C	C<br>C	C<br>C	C<br>C	C<br>C	C<br>C	C<br>C	C<br>C	C<br>C	C<br>C	C<br>C	C<br>C
OAR9_88521505.1	9	88521505	A<br>A	A<br>A	A<br>A	A<br>A	A<br>A	A<br>A	T<br>T	T<br>T	T<br>T	T<br>T	T<br>T	T<br>T	T<br>T	T<br>T	T<br>T	T<br>T	T<br>T	T<br>T	T<br>T	T<br>T
OAR9_88555527.1	9	88555527	C<br>C	C<br>C	C<br>C	C<br>C	C<br>C	C<br>C	A<br>A	A<br>A	A<br>A	A<br>A	A<br>A	A<br>A	A<br>A	A<br>A	A<br>A	A<br>A	A<br>A	A<br>A	A<br>A	A<br>A
OAR9_88619765.1	9	88619765	A<br>A	A<br>A	A<br>A	A<br>A	A<br>A	A<br>A	A<br>A	A<br>A	A<br>A	A<br>A	A<br>A	A<br>A	A<br>A	A<br>A	A<br>A	A<br>A	A<br>A	A<br>A	A<br>A	A<br>A
OAR9_88658895.1	9	88658895	C<br>C	C<br>C	C<br>C	C<br>C	C<br>C	C<br>C	T<br>T	T<br>T	T<br>T	T<br>T	T<br>T	T<br>T	T<br>T	T<br>T	T<br>T	T<br>T	T<br>T	T<br>T	T<br>T	T<br>T
OAR9_88702477.1	9	88702477	G<br>G	G<br>G	G<br>G	G<br>G	G<br>G	G<br>G	C<br>C	C<br>C	C<br>C	C<br>C	C<br>C	C<br>C	C<br>C	C<br>C	C<br>C	C<br>C	C<br>C	C<br>C	C<br>C	C<br>C
s58148.1	9	88749619	G<br>G	G<br>G	G<br>G	G<br>G	G<br>G	G<br>G	A<br>A	A<br>A	A<br>A	A<br>A	A<br>A	A<br>A	A<br>A	A<br>A	A<br>A	A<br>A	A<br>A	A<br>A	A<br>A	A<br>A
OAR9_88784528.1	9	88784528	C<br>C	C<br>C	C<br>C	C<br>C	C<br>C	C<br>C	A<br>A	A<br>A	A<br>A	A<br>A	A<br>C	A<br>A	A<br>A	A<br>A	A<br>A	A<br>A	A<br>A	A<br>A	A<br>A	A<br>A
OAR9_88826213.1	9	88826213	G<br>G	G<br>G	G<br>G	G<br>G	G<br>G	G<br>G	T<br>T	T<br>T	T<br>T	T<br>T	T<br>T	T<br>T	T<br>T	T<br>T	T<br>T	T<br>T	T<br>T	T<br>T	T<br>T	T<br>T
OAR9_88856945_X.1	9	88856946	A<br>A	A<br>A	A<br>A	A<br>A	A<br>A	A<br>A	C<br>C	C<br>C	C<br>C	C<br>C	C<br>C	C<br>C	C<br>C	C<br>C	C<br>C	C<br>C	C<br>C	C<br>C	C<br>C	C<br>C
OAR9_88886896.1	9	88886896	A<br>A	A<br>A	A<br>A	A<br>A	A<br>A	A<br>A	T<br>T	T<br>T	T<br>T	T<br>T	T<br>T	T<br>T	T<br>T	T<br>T	T<br>T	T<br>T	T<br>T	T<br>T	T<br>T	T<br>T
OAR9_88922262.1	9	88922262	G<br>G	G<br>G	G<br>G	G<br>G	G<br>G	G<br>G	C<br>C	C<br>C	C<br>C	C<br>C	C<br>C	C<br>C	C<br>C	C<br>C	C<br>C	C<br>C	C<br>C	C<br>C	C<br>C	C<br>C
OAR9_88974726.1	9	88974726	A<br>A	A<br>A	A<br>A	A<br>A	A<br>A	A<br>A	A<br>A	A<br>A	A<br>A	A<br>A	A<br>A	A<br>A	A<br>A	A<br>A	A<br>A	A<br>A	A<br>A	A<br>A	A<br>A	A<br>A
OAR9_89018996.1	9	89018996	A<br>A	A<br>A	A<br>A	A<br>A	A<br>A	A<br>A	A<br>A	A<br>A	A<br>A	A<br>A	A<br>A	A<br>A	A<br>A	A<br>A	A<br>A	A<br>A	A<br>A	A<br>A	A<br>A	A<br>A

Supplementary Table S3. Chromosome 11 SNP50 Beadchip SNP (genome assembly v1.0) genotypes of affected lambs of flocks A and B. In two affected lambs SNP genotypes were not concordantly homozygous for much of the region (red).

			Affected lamb number																			
SNP ID	Chr	Chr. Posit-ion	1	2	3	4	5	6	7	8	9	10	11	12	13	14	15	16	17	18	19	20
s56248.1	11	27807356	T T	T T	T T	T T	T T	T T	T C	T T	T T	T T	T C	T T	T T	T T	T T	T T	T T	T T	T T	T T
s31301.1	11	27857758	A A	A A	A A	A A	A A	A A	A C	A A	A A	A A	A C	A A	A A	A A	A A	A A	A A	A A	A A	A A
OAR11_27937828.1	11	27937828	C C	C C	C C	C C	C C	C C	T C	C C	C C	C C	T C	C C	C C	C C	C C	C C	C C	C C	C C	C C
s36839.1	11	28072278	G G	G G	G G	G G	G G	G G	G G	G G	G G	G G	G G	G G	G G	G G	G G	G G	G G	G G	G G	G G
s72626.1	11	28404422	C C	C C	C C	C C	C C	C C	T C	C C	C C	C C	T C	C C	C C	C C	C C	C C	C C	C C	C C	C C
OAR11_28473036.1	11	28473036	T T	T T	T T	T T	T T	T T	T T	T T	T T	T T	T T	T T	T T	T T	T T	T T	T T	T T	T T	T T
S49534.1	11	28713152	C C	C C	C C	C C	C C	C C	C C	C C	C C	C C	C C	C C	C C	C C	C C	C C	C C	C C	C C	C C
s49406.1	11	28950668	C C	C C	C C	C C	C C	C C	C C	C C	C C	C C	C C	C C	C C	C C	C C	C C	C C	C C	C C	C C

Supplementary Table S4. Human genes contained in regions of shared homozygosity on ovine chromosomes 9 and 11 (genome assembly version 3.1).

Ovine chromosome containing an extended homozygous region	Gene name	Gene symbol
9	Transmembrane protein 67 (MKS3)	*TMEM67*
9	RNA binding motif protein 12B	*RBM12B*
9	FAM92A1	*FAM92A1*
9	Triple QxxK/R motif containing	*TRIQK*
11	Dishevelled segment polarity protein 2	*DVL2*
11	PHD finger protein 23	*PHF23*
11	GABA(A) receptor associated protein	*GABARAP*
11	CTD nuclear envelope phosphatase 1	*CTDNEP1*
11	Claudin 7	*CLDN7*
11	Elongator acetyltransferase complex subunit 5	*ELP5*
11	Solute carrier family 2, member 4	*SLC2A4*
11	Y box binding protein 2	*YBX2*
11	Translation initiation factor 5A-1	*EIF5A*
11	G protein pathway suppressor 2	*GPS2*
11	Neuralized-like protein 4	*NEURL4*
11	ArfGAP with coiled-coil, ankyrin repeat and PH domains 1	*ACAP1*
11	Potassium channel tetramerisation domain containing 11	*KCTD11*
11	Transmembrane protein 95	*TMEM95*
11	Tyrosine kinase, non-receptor 1	*TNK1*
11	TMEM256-PLSCR3 readthrough	*PLSCR3*
11	Neuroligin 2	*NLGN2*
11	Spermatid maturation 1	*SPEM1*
11	Chromosome 17 open reading frame 74	*C17Orf74*
11	Transmembrane protein 102	*TMEM102*
11	Fibroblast growth factor 11	*FGF11*
11	Cholinergic receptor, nicotinic beta 1	*CHRNB1*
11	Zinc finger and BTB domain containing 4	*ZBTB4*
11	Solute Carrier Family 35 Member G3	*SLC35G3*
11	Polymerase (RNA) II (DNA directed) polypeptide A	*POLR2A*
11	Tumour necrosis factor superfamily member 13	*TNFSF13*
11	Tumour necrosis factor superfamily member 12	*TNFSF12*
11	Sentrin-specific protease 3	*SENP3*

11	Translation initiation factor 4A1	*EIF4A1*
11	CD68 molecule	*CD68*
11	Mannose-P-dolichol utilization defect 1	*MPDU1*
11	SRY– box 15	*SOX15*
11	Fragile X mental retardation, autosomal homolog 2	*FXR2*
11	Spermidine/Spermine N1-acetyltransferase family member 2	*SAT2*
11	Sex hormone binding globulin	*SHBG*
11	ATPase NA/K transporting, beta 2 polypeptide	*ATP1B2*
11	Tumour protein P53	*TP53*
11	WRAP53	*WRAP53*
11	Ephrin-B3	*EFNB3*
11	Dynein, axonemal, heavy chain 2	*DNAH2*
11	N(alpha)-acetyltransferase 38, NatC auxiliary subunit	*NAA38*
11	Lysine (K)-specific demethylase 6B	*KDM6B*
11	Transmembrane protein 88	*TMEM88*
11	cytochrome b5 domain containing 1	*CYB5D1*
11	Chromodomain helicase DNA binding protein 3	*CHD3*
11	Potassium channel, voltage gated subfamily A regulatory beta subunit 3	*KCNAB3*
11	Trafficking protein particle complex 1	*TRAPPC1*
11	Centrobin, centrosomal BRCA2 interacting protein	*CNTROB*
11	Guanylate cyclise 2D, membrane (retina specific)	*GUCY2D*
11	Arachidonate 15-lipoxygenase, type B	*ALOX15B*
11	Arachidonate 12-lipoxygenase, 12R type	*ALOX12B*

Supplementary Table S5. Summary of sequence variants identified in exons located in the regions of shared homozygosity on ovine chromosomes 9 and 11 (genome assembly version 1) in affected lambs.

**Gene**	**Homo-zygous/ Hetero -zygous**	**Nucleotide nomenclature**	**Mutation type**	**Amino acid change**	**Protein change type**	**Sift prediction**
TNK1	Hom	Chr11:26699152C>G	substitution	PRO>ALA	Missense	Tolerated
ZBTB4	Hom	Chr11:26766513G>A	substitution	GLY>SER	Missense	Tolerated
TNFSF12	Hom	Chr11:26842625T>G	substitution	LEU>ARG	Missense	Tolerated
TNFSF12	Hom	Chr11:26842745T>C	substitution	LEU>SER	Missense	Tolerated
WRAP53	Hom	Chr11:26960401-26960402G-[]	deletion		Frame shift	-
TMEM67	Hom	Chr9:87966948T>A	substitution	ILE>ASN	Missense (I681N)	Non-tolerated
TMEM67	Hom	Chr9:87966930T>G	substitution	ILE> SER	Missense (I687S)	Non-tolerated

Supplementary Table S6. Outcome from using A-GVGD, SIFT and Polyphen to determine whether the predicted missense amino acid changes in *TMEM67* would be tolerated or would potentially affect protein function.

SNP	A-GVGD	SIFT	Polyphen
I681N	Predicted deleterious	Predicted to affect protein function	Predicted to probably be damaging
I687S	Predicted deleterious	Predicted to affect protein function	Predicted to probably be damaging

Supplementary Table S7. Genotyping results from 40 sheep; correlation with phenotype. NT= Not tested; carriers highlighted in red.

Flock B				
**Observed status**	**Sex**	**Tag number**	**Restriction digest result**	**Gave birth to PKD lamb**
Carrier (confirmed)	Ewe	1040	Carrier	Yes
Carrier (confirmed)	Ewe	34	Carrier	Yes
Carrier (confirmed)	Ewe	33	Carrier	Yes
Carrier (confirmed)	Ewe	5011	Carrier	Yes
Unaffected	Ewe	6003	WT	No
Unconfirmed	Ewe	6016	Carrier	NT
Unaffected	Ewe	6023	WT	No
Unaffected	Ewe	7004	WT	No
Carrier (confirmed)	Ewe	7006	Carrier	Yes
Unconfirmed	Ewe	7008	Carrier	NT
Unaffected	Ewe	7013	WT	No
Unaffected	Ewe	7016	WT	No
Unaffected	Ewe	8002	WT	No
Unconfirmed	Ewe	8003	Carrier	NT
Unconfirmed	Ewe	8006	Carrier	NT
Unconfirmed	Ewe	8007	Carrier	NT
Unaffected	Ewe	8008	WT	No
Unaffected	Ewe	8009	WT	No
Unaffected	Ewe	8010	WT	No
Unaffected	Ewe	8016	WT	No
Unconfirmed	Ewe	9003	Carrier	NT
Unaffected	Ewe	9004	WT	No
Unaffected	Ewe	9007	WT	No
Unaffected	Ewe	9009	WT	No
Unaffected	Ewe	9010	WT	No
Unaffected	Ewe	9011	WT	No
Unconfirmed	Ewe	9018	Carrier	NT
Carrier (confirmed)	Ram	6005	Carrier	Yes
Carrier (confirmed)	Ram	6093	Carrier	Yes
Carrier (confirmed)	Ram	6026	Carrier	Yes
Unaffected	Ram	8031	WT	No
Unconfirmed	Ram	8034	Carrier	NT
Unconfirmed	Ram	8039	Carrier	NT
Unconfirmed	2009 lamb	C	Carrier	NT
Affected	2009 lamb	D	Hom mutant	-
Affected	2009 lamb	E	Hom mutant	-
Affected	2009 lamb	F	Hom mutant	-
Affected	2009 lamb	G	Hom mutant	-
Unaffected	2009 lamb	I	WT	No
Unaffected	2009 lamb	J	WT	No

Supplementary Table S8. Primer sequences for PCR and sequencing of exon 20 of ovine *TMEM67*. Sequences are presented 5’ to 3’. TM= Melting temperature

**Primer name**	**Primer sequence**	**TM**
TMEM67-fE	AATGGTGGTTCCAGGTCTGA	60
TMEM67-rE	TTGCGTTTGTCATTCTCAGG	59

Supplementary Figures

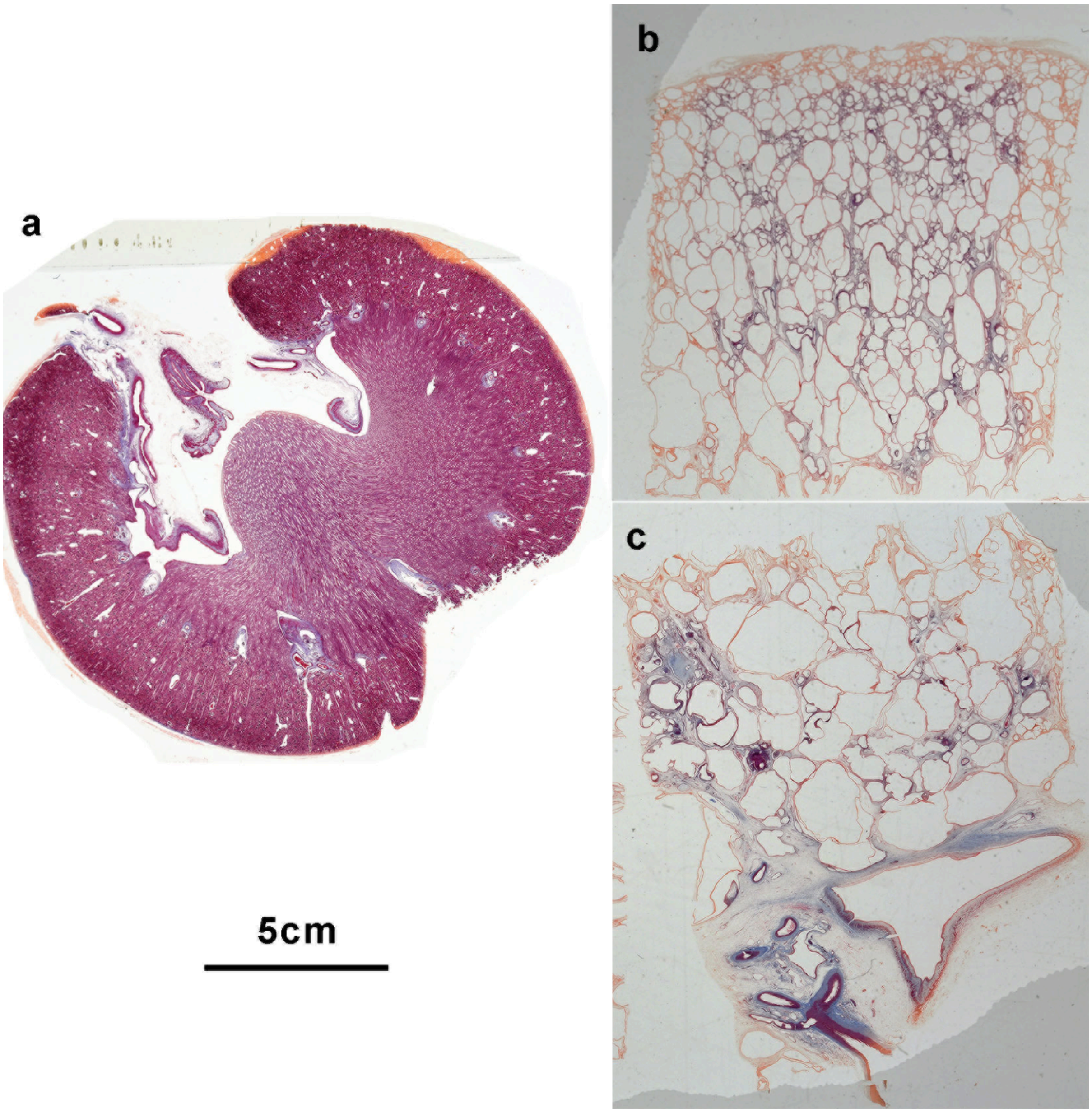

Supplementary Figure S1. Cysts and fibrosis are present throughout the entire kidney: Photographs of (a) unaffected kidney section stained with Periodic-Acid Schiff (PAS), and (b, c) sections from an affected kidney which was cut in half to fit on a microscope slide. (b) Representative image of the cortical region and, (c) the inner medullary region. This demonstrates that cysts were present throughout the kidney and involved both the cortex and medulla regions. The images in (a-c) were acquired at the same magnification.

```
Hum.   1   MATRGGAG-VAMAVWSLLSARAVTAFLLLFLPRFLQAQTFSFPFQQPEKC
           ||| |    ||||  | |||  || |||| |||  ||||| ||| ||| |
Ovine  1   MATGGRGRVVAMAARSTLSAESVTLFLLLVLPRVSQAQTFFFPFRQPETC
                                      ||     |  | |   ||||  |
Danio      ------------------------IIILLKISVFSCQQ-FSISFRQPSDC

Hum.   50  DNNQYFDISALSCVPCGANQRQDARGTSCVCLPGFQMISNNGGPAIICKK
             |||||||||||| |||||||||||||||| ||||||||||||  | |||
Ovine  51  GHNQYFDISALSCVACGANQRQDARGTSCVCSPGFQMISNNGGRDITCKK
           | | ||||| |||| || ||      |  | |  |        |  | |
Danio      GVNSYFDISSLSCVKCGSNQNRECIGLACECQAGSRVLVS-NGVSILCQQ

Hum.   100 CPENMKGVTEDGWNCISCPSDLTAEGKCHCPIGHILVERDINGTLLSQAT
           ||| ||||||||||||||||  |||||||||| ||||||| |||||||||
Ovine  101 CPEHMKGVTEDGWNCISCPGGLTAEGKCHCPTGHILVERNVNGTLLSQAT
           ||     || ||  || |||     ||| || |  ||||  ||  |  |
Danio      CPDADQVVTQDGYECIRCPGTVGKDGKCQCPEGSVLVERDGNGNPLIEAR

Hum.   150 CELCDGNENSFMVVNALGDRCVRCEPTFVNTSRSCACSEPNILTGGLCFS
           |||||  |||| | |||| ||||||||| |||||||||||||||||||||
Ovine  151 CELCDESENSFTVANALGNRCVRCEPTFINTSRSCACSEPNILTGGLCFS
           |  |      |      | || ||   | | | || |   | |
Danio      CQACSATDPAFSAPDMKGNRCERCGDSFINSSQSCVCGTSNTL=======

Hum.   200 STGNFPLRRISAARYGEVGMSLTSEWFAKYLQSSAAACWVYANLTSCQAL
           |||||||| |||||||| | | || |||||||||||||||||| |||||||
Ovine  201 STGNFPLRMISAARYGELGISFTSAWFAKYLQSSAAACWVYGNLTSCQAV
                                  ||||     ||||||    | | ||
Danio      ==================TLPVQSAWFSSFMYSSAAACLLFSNRTACQSL

Hum.   250 GNMCVMNMNSYDFATFDACGLFQFIFENTAGLSTVHSISFWRQNLPWLFY
           ||||||||| ||  ||||||||| |||||| | |||| ||||||||||||
Ovine  251 GNMCVMNMNYYDSSTFDACGLFQVIFENTAALGTVHSVSFWRQNLPWLFY
           ||||||||    |   | |||   |   ||| |     | || ||||| |
Danio      GNMCVMNMHSSSSINNDVCGLYNTIYRTTAAQGSSQDISYWRTNLPWLYY

Hum.   300 GDQLGLAPQVLSSTSLPTNFSFKGENQNTKLKFVAASYDIRGNFLKWQTL
           ||||||||| || | |||||||||||||||||||||||| ||||||||||
Ovine  301 GDQLGLAPQILSTTPLPTNFSFKGENQNTKLKFVAASYDVRGNFLKWQTL
           ||| | | | |   |||  || ||   ||     || | ||| || |
Danio      GDQPGMASQALQNEPLPVGFSFKGAQKNTNIELRAAVYSVRGEFLRWESV
```

```
Hum.   350 EGGVLQLCPDTETRLNAAYSFGTTYQQNCEIPISKILIDFPTPIFYDVYL
           ||| ||||||||||||||||||||||| ||||| ||| ||||| ||||
Ovine  351 EGGILQLCPDTETRLNAAYSFGTTYQQSCEIPIPKILTDFPTPTFYDVYL
             | ||||||   |  || |||||| | |       ||  | | |||| |
Danio      GRGNLQLCPDIVARIDAAFSFGTTYKQECVLSVSELLTGYPEPLFYDVFL

Hum.   400 EYTDENQHQYILAVPVLNLNLQHNKIFVNQDSNSGKWLLTRRIFLVDAVS
           ||||||||||| ||||||||||| | |||||| ||||||||||||||  |
Ovine  401 EYTDENQHQYIWAVPVLNLNLQHKKMFVNQDSSSGKWLLTRRIFLVDTLS
                 |     ||| || | |    |||| |   || ||||  ||||||
Danio      GTIQNSQDNRLLAVPLLNANQQFNGQFVNQGSDMSKWFLTRRLMLVDTLS

Hum.   450 GRENDLGTQPRVIRVATQISLSVHLVPNTINGNIYPPLITIAYSDIDIKD
           ||||||| ||| |||||||||| |||||| ||||||||||| ||| ||||
Ovine  451 GRENDLGSQPRLIRVATQISLSIHLVPNTKNGNIYPPLITIVYSDVDIKD
           |||    | |  ||||  |     |||||  |  ||||    |||  |||
Danio      GREKSISSSPVVIRVASDIKIGFQLVPNTQKGQVYPPLMSVAYSDIQIKD

Hum.   500 ANSQSVKVSFSVTYEMDHGEAHVQTDIALGVLGGLAVLASLLKTAGWKRR
            ||||||||||||||||  || |||||||||||||||| |||||||||||
Ovine  501 PNSQSVKVSFSVTYEMDQREAQVQTDIALGVLGGLAVLSSLLKTAGWKRR
           |  | | ||||| |||        |||||||||| ||  |||||| ||||
Danio      PSTQTVTVSFSVNYEMRQLDSLKKTDIALGVLGGVAVVHSLLKTASWKRR

Hum.   550 IGSPMIDLQTVVKFLVYYAGDLANVFFIITVGTGLYWLIFFKAQKSVSVL
           ||||||||||| ||||||||||||||||||||||||||||||||||||||
Ovine  551 IGSPMIDLQTVTKFLVYYAGDLANVFFIITVGTGLYWLIFFKAQKSVSVL
           | || ||| |  |||| |||||||||||||||||||||||||||  ||||
Danio      IASPLIDLETIMKFLVFYAGDLANVFFIITVGTGLYWLIFFKAQLFVSVL

Hum.   600 LPMPIQEERFVTYVGCAFALKALQFLHKLISQITIDVFFIDWERPKGKVL
           || | ||||||||||| ||||||||||||||||||| |||||||||||||
Ovine  601 LPVPAQEERFVTYVGCGFALKALQFLHKLISQITIDIFFIDWERPKGKVL
           || |||||||| |||| ||||  |||||   |   | |||||||| ||
Danio      LPLPAQEERFVVYVGCAFALKTVQFLHKIFVQLSVDVFFIDWERPRGKTT

                                         *     *
Hum.   650 KAVEGEGGVRSATVPVSIWRTYFVANEWNEIQTVRKINSLFQVLTVLFFL
           |||||||||||||||||||||||||||||||||||||| ||||| |||||
Ovine  651 KAVEGEGGVRSATVPVSIWRTYFVANEWNEIQTVRKINPLFQVLTVLFLL
           | ||| |   |   ||||||||||||||||||| ||||| |||   ||||
Danio      KMVEGSGETKSQASPVSIWRTYFVANEWNEIQTIRKINPTFQVMAVLFFL
```

```
Hum.   700 EVVGFKNLALMDSSSSLSRNPPSYIAPYSCILRYAVSAALWLAIGIIQVV
           ||||||||||||||||||| ||||||||| ||||||| |||| ||||| |
Ovine  701 EVVGFKNLALMDSSSSLSRSPPSYIAPYSRILRYAVSSALWLVIGIIQIV
           ||||| |||| |||| | ||   |  ||| |||| |  | || || ||
Danio      EVVGFSNLALRDPSSNLNRSAEEYTPPYSLILRYGVATAMWLCIGLIQMI

Hum.   750 FFAVFYERFIEDKIRQFVDLCSMSNISVFLLSHKCFGYYIHGRSVHGAD
           || |||||||||||||||||| |||||| |||| ||||||||||||||||
Ovine  751 FFGVFYERFIEDKIRQFVDLCCMSNISVLLLSHRCFGYYIHGRSVHGAD
           || || ||| |||||||||||  |||||||||||||||||||||||||||
Danio      FFTVFHERFVEDKIRQFVDLCSISNISVLLLSHRCFGYYIHGRSVHGAD

Hum.   800 TNMEEMNMNLKREAENLCSQRGLVPNTDGQTFEIAISNQMRQHYDRIHEL
           ||||||| |||||||||||||||||||||||| |||| |||||||||||
Ovine  801 TNMEEMNRNLKREAENLCSQRGLVPNTDGQTFQIAISSQMRQHYDRIHET
           ||| ||| |||||||||| |||| || | ||||| |    |  |||| |
Danio      TNMDEMNTNLKREAENLCGQRGLLPNSDTQTFQISITNRLRAQYDRIIEP

Hum.   850 LIRKNGPARLLSSSASTFEQSIKAYHMMNKFLGSFIDHVHKEMDYFIKDK
           | |||||||||||| |||||| |||| |||||||||||||||||||||||
Ovine  851 LTRKNGPARLLSSSGSTFEQSVKAYHTMNKFLGSFIDHVHKEMDYFIKDK
             |  || ||        ||| ||||||| |||| ||| | ||||  |||
Danio      ISRRRGPSRLVDATANPCEQSTKAYHTMNRFLGSVIDHAHREMDYIVKDK

Hum.   900 LLLERILGMEFMEPMEKSIFYNDEGYSFSSVLYYGNEATLLIFDLLFFCV
           ||||||||||||||||||||||||||||| |||||||||||| || ||||
Ovine  901 LLLERILGMEFMEPMEKSIFYNDEGYSFSNVLYYGNEATLLIYDLMFFCV
           || ||  |||| ||  ||||||||  ||  || ||||  ||| |  ||||
Danio      LLFERVIGMEFIEPLDKSIFYNDENHSFTDVLFYGNEGVLLIFDTLFFCV

Hum.   950 VDLACQNFILASFLTYLQQEIFRYIRNTVGQKNLASKYLVDQRFLI*
           |||||||| || ||||||||||| ||| ||||||||| ||||||||
Ovine  951 VDLACQNFVLAAFLTYLQQEIFRFIRNAVGQKNLASKTLVDQRFLI*
           |||  ||| ||| |||||| ||| |||  |  ||| ||||| ||||
Danio      VDLGKQNFILAAVLTYLQQMIFRLIRNGLGRRNLANKTLVDKRFLI*
```

Supplementary Figure S2. Alignment of human, ovine and zebrafish predicted MKS3 amino acid sequences. Asterisks above the sequence show the positions of the mutations (not identified here). The sequences aligned were human (Hum.), sheep (Ovine), and zebrafish (Danio). Alternative exon usage (===) was observed in zebrafish, and this sequence also lacked the N-terminal end of the protein (---). Amino acid identity observed to occur between MKS3 in two species is shown with (|).

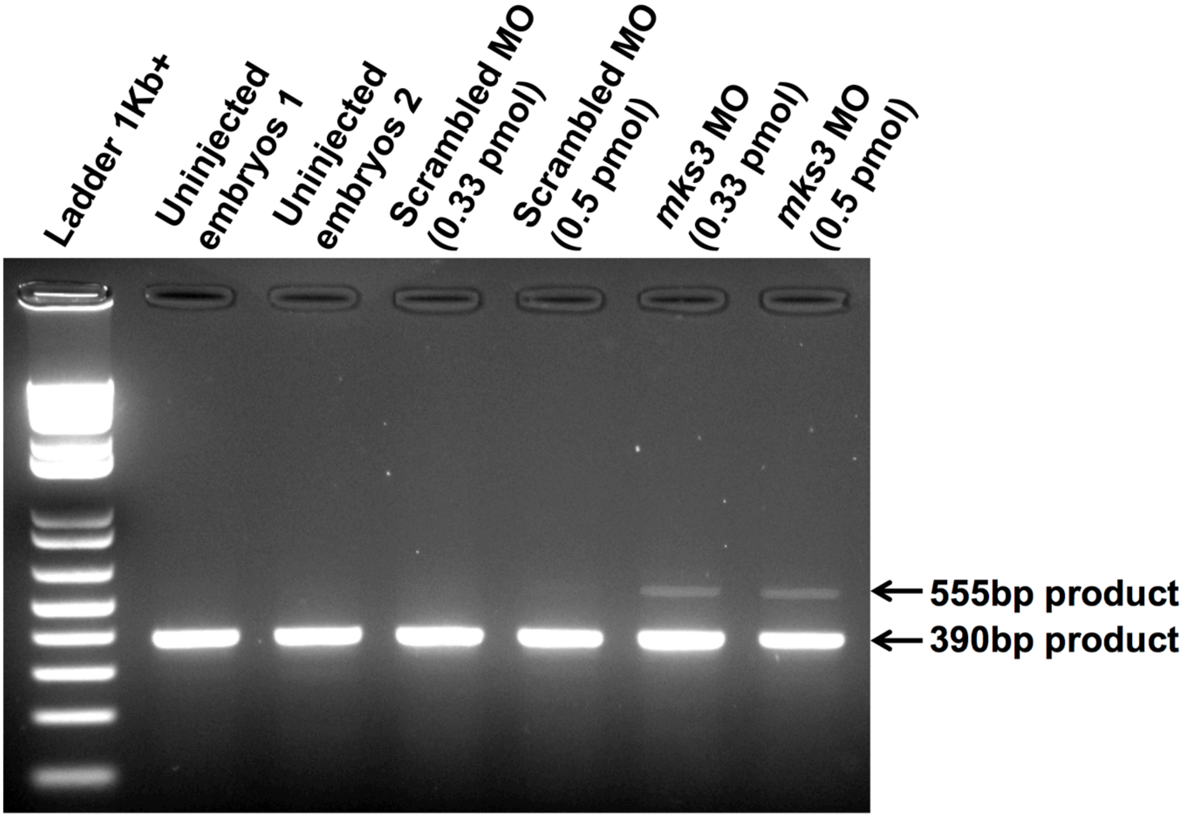

Supplementary Figure S3. Confirmation of altered splicing of zebrafish *tmem67* transcripts as a result of microinjection of zebrafish embryos with *tmem67splice* morpholino (MO). RT-PCR of pooled zebrafish embryos (n=20) at 72 hpf, uninjected, or injected with 0.33 pmol or 0.5 pmol of either “scrambled MO”, or “*mks3* MO” targeting tmem67 transcripts, revealed an additional larger RT-PCR product (555 bp) in “*mks3* MO” injected embryos, compared to uninjected or scrambled MO injected control embryos, which only had a normal RT-PCR product of 390 bp. The 555 bp PCR product was the expected size of *tmem67* transcripts that were affected by the *tmem67splice* MO.

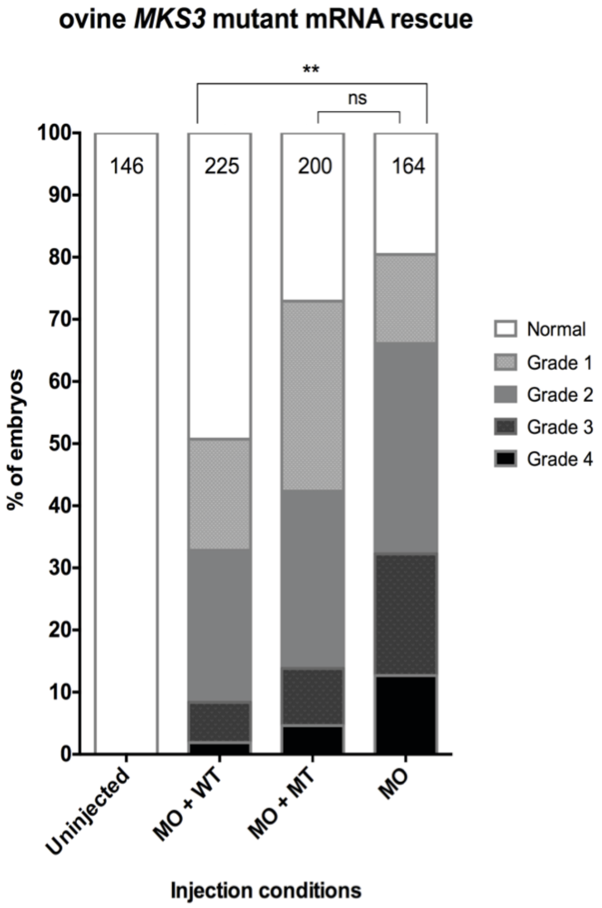

Supplementary Figure S4. Graph showing the phenotypic rescue of *mks3* morphant embryos. Zebrafish embryos were co-injected at the 11-12 somite stage of development with ovine *OaMKS3* mRNA plus MO and scored on direct morphological appearance of the embryos, which were graded in terms of the severity of phenotype as indicated, from normal to Grade 4. Co-injection of MO plus ovine wild type *OaMKS3* mRNA (MO+WT) resulted in a significant restoration of the normal phenotype in the morphant embryos compared with MO-injected embryos (**p < 0.0001, 2-tailed Fisher Exact test), whereas the co-injection of MO plus ovine mutant *OaMKS3(I681N;I687S)* mRNA (MO+MT) was insignificant at the level of 0.01 (p = 0.035) in restoring the normal phenotype in the morphant embryos compared with MO-injected embryos. The number shown at the bottom of each bar states the total number of embryos used in each injection condition. Abbreviations: MO, morpholino; WT, Wild Type *Tmem67*/*OaMKS3* mRNA; MT, Mutant *Tmem67/OaMKS3(I681N;I687S)* mRNA; ns, not significant.

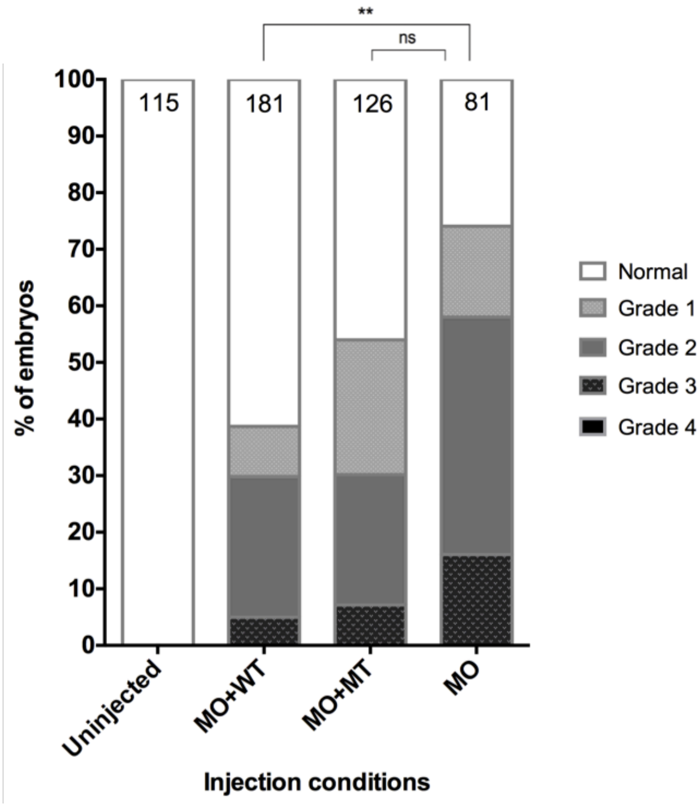

Supplementary Figure S5. Percentages of 60-72 hpf rescued zebrafish embryos (with 50 ng mRNA) in different morphological grades using direct observation under a bright-field microscope. Co-injection of MO plus ovine wild type *OaMKS3* mRNA (MO+WT) resulted in a significant restoration of the normal phenotype in the morphant embryos compared with MO-injected embryos (**p-value < 0.0001, 2-tailed Fisher Exact test), whereas the co-injection of MO plus ovine mutant *OaMKS3(I681N;I687S)* mRNA (MO+MT) did not significantly restore the normal phenotype in the morphant embryos compared with MO-injected embryos (p = 0.5567). The number located at the top of each bar indicates total number of embryos used in each injection condition including deaths of the grade 4 morphants. Abbreviations: MO, morpholino; WT, *OaMKS3* mRNA; MT, *OaMKS3(I681N;I687S)* mRNA; ns, not significant.

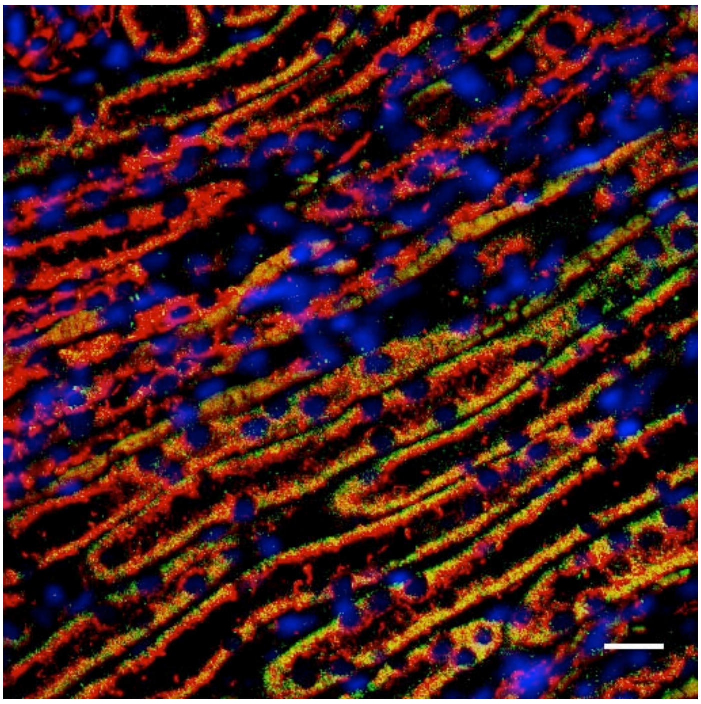

Supplementary Figure S6. Meckelin expression in unaffected lamb kidney epithelial cells. Immunofluorescent localization of acetylated α-tubulin (red) and meckelin (green) expression in renal tubule epithelial cells of an unaffected newborn lamb kidney. Cell nuclei were stained with DAPI. Scale bar = 50 µm.

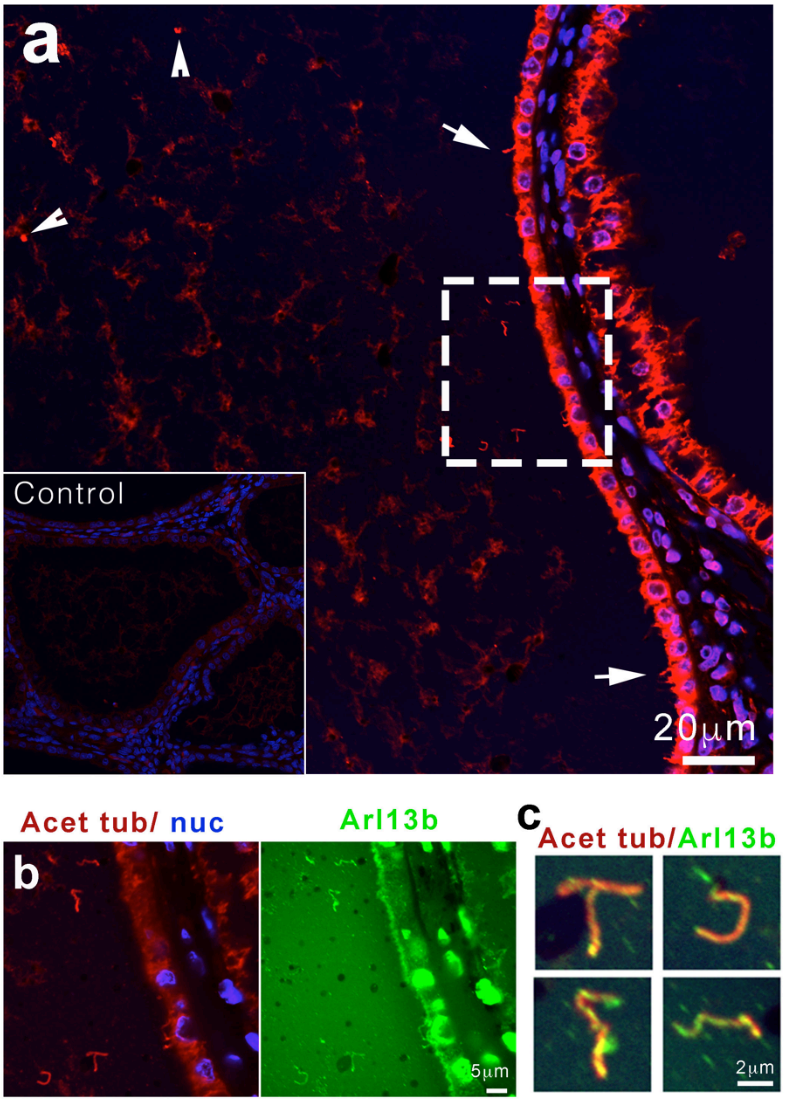

Supplementary Figure S7. Ciliary fragments in cyst lumen. (a) Confocal z-slice (slice thickness 0.6um) of an affected lamb kidney section labelled with acetylated α-tubulin (red) and Hoechst nuclear label (blue). Arrows indicate primary cilia associated with cyst epithelia; arrowheads indicate tubulin-positive structures, possibly ciliary fragments. White dashed box contains several acetylated α-tubulin-positive structures within the cyst fluid. Fragments were not associated with cyst epithelia. The lighter red material in the cyst lumen represents autofluorescence of the luminal contents. (b) High magnification images of dashed white box: acetylated α-tubulin (red), nuclei (blue) and arl13b (green). Fragments are immunopositive for both ciliary markers and are not associated with any nuclei. (c) Ciliary fragments at higher magnification showing co-localisation of acetylated α-tubulin and arl13b.

Supplementary Movie M1. Primary cilia fragments located within the cyst lumen. Z-series through 9μm kidney section labelled with acetylated α-tubulin (red) to demonstrate primary cilia on cyst epithelia and fragments located within the cyst lumen. Nuclei (blue) are labelled with Hoechst. Image series was captured at 400x magnification using a 1.3NA lens, and at 2048 x 2048 pixels.

Supplementary Information

Additional information on the homozygosity mapping in affected lambs:
The homozygous region on chromosome 9 (OAR9v1.0: 87942007-89018996; OAR9v3.1: 83056186-884133462) contained 26 consecutive homozygous SNPs (in genome assembly v1.0) in all six affected lambs used for screening of flock A, and 13 of 14 of the affected lambs used for screening in flock B (Fig 3A and Supplementary Tables 1-2), and encompassed five predicted genes (Supplementary Table 4). For the SNPs on chromosome 9, homozygosity was observed in all SNPs, except OAR9_88126600.1, OAR9_88387736.1, and OAR9_88784528.1, which were heterozygous in one affected lamb in flock B. This may be explained if the region of shared homozygosity had sustained a recombination event near one of the SNPs (OAR9_88113366.1) in this one affected lamb. Note that for 19/26 SNPs the homozygous alleles identified on chromosome 9 in flock B showed a different set of genotypes to those in flock A. There were, nevertheless, two sets of two consecutive homozygous SNPs (OAR9_88113366.1 and OAR9_88126600.1; OAR9_88974726.1 and OAR9_89018996.1), that shared identical genotypes in flocks A and B. Our interpretation of these findings is that a recombination event had occurred near the *TMEM67* gene in one of the sheep breeds during its establishment as a new sheep breed. Therefore, even though there is concordant homozygosity (though not necessarily identical genotypes) of the 26 SNPs (or 25 SNPs in genome assembly v3.1) in flocks A and B, the genotypes in flock A are predominantly different from those in flock B. Nevertheless, two consecutive homozygous

SNP genotypes (OAR9_88113366.1 and OAR9_88126600.1), located near the *TMEM67* locus, were concordant and identical in both flocks A and B.

A second homozygous region was located on chromosome 11 in flocks A and B (OAR11v1.0: 27807356-28950668; OAR11v3.1: 26571629-27285522), which encompassed 51 predicted genes (Supplementary Table 4), and contained 8 consecutive concordant homozygous SNPs (in genome assembly v1.0) (Fig 3B and Supplementary Tables 1, 3) in all six affected lambs used for screening of flock A, and 12 of the 14 affected lambs used for screening in flock B. Two affected lambs of flock B were heterozygous at 4 of the 8 SNPs in the homozygous region on chromosome 11 (Supplementary Table S3).

Additional information on the sequence variants identified:

Six missense variants and one frame-shift variant were identified following analysis of sequence variants present in the homozygous regions, particularly focusing on the sequence variants present in exons of four genes on chromosome 9, and 51 genes on chromosome 11. Each of these variants is predicted to result in a change in the encoded protein. Using the 'Sorting Intolerant From Tolerant' (SIFT) algorithm, four of the identified sequence variants would be tolerated, while two genes, *WRAP53* and *TMEM67*, contained potentially pathogenic variants. Compound heterozygous mutations in *WRAP53* have previously been reported in human patients, resulting in dyskeratosis. Therefore, although the *WRAP53* frameshift variant was homozygous in the affected lamb's DNA, abnormalities in human patients with this mutation were inconsistent with the abnormalities present in the affected lambs.
